# Mapping the Reliability-Readability Gap in the Education of Patients With Age-Related Macular Degeneration Across 6 Large Language Models: Comparative Evaluation Study

**DOI:** 10.2196/91016

**Published:** 2026-07-28

**Authors:** Zhili Lu, Haixing Cao, Cong Ma, Jin Zheng, Xiang Ma

**Affiliations:** 1 Department of Ophthalmology, First Affiliated Hospital of Dalian Medical University, 222 Zhongshan Road, Dalian City, Liaoning Province, Dalian, Liaoning, 116011, China, 86 18098876399

**Keywords:** age-related macular degeneration, health literacy, information quality, large language models, patient education

## Abstract

**Background:**

Artificial intelligence–generated health information is increasingly used by patients, but its reliability, visible transparency indicators, and readability remain uncertain in specialized ophthalmic conditions such as age-related macular degeneration (AMD).

**Objective:**

This study aimed to evaluate and compare the informational reliability, visible transparency indicators, overall quality, and readability of responses generated by 6 publicly accessible large language models (LLMs) to AMD-related patient-facing prompts under a zero-shot, single-turn prompting scenario.

**Methods:**

Thirty English-language AMD-related prompts were curated from Google Trends, the 2023 Chinese AMD guideline, and the 2025 American Academy of Ophthalmology Preferred Practice Pattern. Chinese guideline–derived prompts were translated and reviewed before model querying. Each finalized prompt was entered verbatim into ChatGPT-5.1-auto, DeepSeek-v3.2, Gemini-2.5-Flash-Thinking, Grok 4, Claude-Sonnet 4.5, and Qwen3-Max between October 10 and November 25, 2025. Two senior ophthalmologists (ZL and XM) blinded to model identity independently scored all responses using DISCERN, Ensuring Quality Information for Patients (EQIP), Global Quality Scale, and Journal of the American Medical Association benchmark criteria, with adjudication for disagreements. Readability was assessed using 6 standard formulas against a sixth-grade benchmark. Between-model differences were analyzed using Friedman tests with Holm-adjusted pairwise comparisons.

**Results:**

A total of 180 responses were analyzed. Interrater agreement was substantial to near-perfect across reliability instruments (κ=0.72‐0.97). No model met the recommended sixth-grade readability target. Grok 4 achieved the highest scores on reliability-related instruments, including DISCERN (mean 46.40, SD 7.43) and EQIP (mean 74.33, SD 9.07), whereas DeepSeek-v3.2 generated the most readable responses, with the highest Flesch Reading Ease Score (mean 48.23, SD 9.16) and lowest Flesch-Kincaid Grade Level (mean 9.95, SD 1.87). Significant between-model differences were observed across all reliability and readability metrics (all *P*<.001).

**Conclusions:**

Under zero-shot, single-turn prompting conditions, the evaluated public LLMs showed substantial model-dependent differences in AMD-related patient education quality and readability. No model met the sixth-grade readability benchmark, including those with comparatively stronger reliability performance. These findings support clinician oversight, readability optimization, and further evaluation before LLM-generated AMD information is used directly in patient-facing settings.

## Introduction

Age-related macular degeneration (AMD) is a leading cause of irreversible vision loss in older adults worldwide [1]. Management of this condition, which encompasses intravitreal anti–vascular endothelial growth factor (VEGF) therapy, nutritional supplementation, and lifestyle modification, is complex and requires the informed and active participation of the patient [2]. Effective patient education is therefore crucial, yet often constrained by limited clinical time, driving patients to seek information online [3]. Online patient education materials in ophthalmology have been shown to vary substantially in readability, accountability, and suitability, and many exceed recommended reading levels. Similar concerns have also been reported for artificial intelligence (AI)–generated ophthalmology patient education materials [3-5]. These findings support the need to evaluate whether large language model (LLM)–generated AMD information is both reliable and understandable for patients.

The rapid emergence of LLMs as conversational agents presents a transformative opportunity for scalable health communication. Tools such as OpenAI’s ChatGPT are accessed by hundreds of millions of users and demonstrate an ability to simplify medical text toward lower reading levels upon explicit instruction [6,7]. In ophthalmology, early evaluations suggest that LLMs can generate generally coherent responses to common patient queries about conditions such as AMD and glaucoma, aligning with their “impressive but mixed” performance across broader medical contexts [3,8].

However, 2 critical and interrelated barriers impede their direct clinical deployment for patient education. First, readability remains persistently excessive and should be interpreted within the broader framework of health literacy. Health literacy determines whether patients can obtain, understand, evaluate, and use medical information in decisions about their care. The National Institutes of Health, the US Department of Health and Human Services, and the American Medical Association have recommended that patient education materials be written at approximately a sixth-grade reading level [9]. This issue is particularly relevant to AMD, which predominantly affects older adults and may impose additional reading burdens because of central visual impairment. Nevertheless, most ophthalmic education content, including traditional handouts and website-based materials, far exceeds this recommendation [4,10,11]. This concern also extends to AMD-specific patient education materials and AI-generated ophthalmology patient education materials, which frequently remain above recommended reading levels [4,5]. Whether improved readability can be achieved without compromising informational reliability remains an empirical question in the education of patients with AMD. Therefore, this study jointly evaluated readability and reliability rather than treating readability as an isolated linguistic outcome.

Current evaluations in ophthalmology, while noting issues of factual omission or nuance, have not systematically quantified this trade-off using standardized, multidimensional reliability instruments (eg, DISCERN and Ensuring Quality Information for Patients [EQIP]) alongside rigorous readability metrics [4,12,13].

Crucially, the performance landscape of state-of-the-art LLMs remains unmapped in specialized clinical domains such as AMD. Existing multimodel comparisons are either generic or limited in scope, leaving a significant gap in understanding how newer, advanced models (eg, Grok 4, Claude-Sonnet 4.5, and DeepSeek-v3.2) perform relative to each other [14]. Furthermore, assessments conducted under a “zero-shot”– prompting scenario, which best simulates a naive patient’s initial and unoptimized query, are essential to establish a realistic performance baseline but are seldom the focus [15,16].

Therefore, to move from generic caution toward actionable evidence, this study aims to (1) conduct a systematic, head-to-head evaluation of 6 leading LLMs on a curated set of AMD patient–facing prompts; (2) simultaneously quantify output quality using 4 established reliability tools (DISCERN, EQIP, Global Quality Scale [GQS], and Journal of the American Medical Association [JAMA] benchmark criteria) and 6 readability formulas; and (3) explicitly analyze the interplay—or trade-off—between reliability and readability under a minimalist, real-world-query simulation. By establishing this comprehensive performance atlas, we provide the empirical foundation necessary for the informed selection, targeted optimization, and safe, human-supervised integration of LLMs into ophthalmic patient care.

## Methods

### Prompt Development and Selection

A set of 30 English-language prompts on AMD was formulated through a multistep process (Textbox 1). First, an analysis of global Google Trends data from October 10, 2020, to October 10, 2025, was conducted based on the MeSH term “Age-Related Macular Degeneration” to identify frequently used patient search queries. This yielded 25 high-frequency search phrases. Second, 8 clinically focused prompts were extracted from the “Evidence-Based Guidelines for Diagnosis and Treatment of Age-Related Macular Degeneration in China (2023)” [17]. For these Chinese guideline–derived prompts, the initial English translation was performed by ZL immediately after extraction. The translated prompts were then reviewed and validated by XM for semantic equivalence, ophthalmic terminology, and consistency with English clinical expression. Subsequently, 10 topic prompts were extracted from the 2025 American Academy of Ophthalmology “Age-Related Macular Degeneration Preferred Practice Pattern” [18]. Finally, from these 43 initial prompts, irrelevant or duplicate entries were removed, resulting in 30 unique English-language prompts (Textbox 1). These finalized English-language prompts were entered verbatim into the 6 evaluated LLMs without modification, and the responses were collected for further analysis. The final sample size of 30 prompts was selected a priori to provide a representative cross-section of common public-search, guideline-derived, and clinically relevant AMD information needs while preserving the feasibility of manual-blinded evaluation by ophthalmologist raters.

Textbox 1. **Curated prompt set for evaluating large language models in the education of patients with age-related macular degeneration (n=30).**

Google Trends data of the 25 most significant keywords queried globally for age-related macular degeneration between 2020 and 2025:

1. macular degeneration icd 10

2. neovascular age-related macular degeneration

3. neovascular

4. exudative age-related macular degeneration

5. nonexudative age-related macular degeneration

6. age-related macular degeneration treatment

7. macular degeneration treatment

8. what is macular degeneration

9. age-related macular degeneration

10. exudative macular degeneration icd 10

11. nonexudative age-related macular degeneration icd 10

12. choroidal neovascularization

13. retinopathy

14. age-related macular degeneration symptoms

Evidence-based guidelines for diagnosis and treatment of age-related macular degeneration in China (2023):

15. What is the preferred diagnostic modality for neovascular AMD?

16. Should nutritional supplements be recommended for patients with early to intermediate AMD?

17. How should patients with AMD who exhibit a suboptimal initial response to anti-VEGF therapy be managed?

18. How should persistent pigment epithelial detachment after anti-VEGF therapy in AMD patients be managed?

19. How should “nonexudative” macular neovascularization (MNV) detected by OCTA in AMD patients be managed?

20. What is the appropriate follow-up protocol for patients with AMD?

Age-related macular degeneration preferred practice pattern (2025 American Academy of Ophthalmology):

21. AMD Definition and Classification

22. AMD Epidemiology and Risk Factors

23. AMD Diagnosis and Monitoring

24. Early and Intermediate AMD Management

25. Neovascular AMD Anti-VEGF Therapy

26. Geographic Atrophy in AMD

27. AMD Treatment Protocols

28. AMD Treatment Complications

29. AMD Patient Management

30. AMD Socioeconomic Considerations

Note on prompt curation: From the initial 25 Google Trends queries, 11 were excluded: “icd 10,” “exudative macular degeneration,” “exudative,” “nonexudative,” “amd,” “dry macular degeneration,” “dry age-related macular degeneration,” “wet age-related macular degeneration,” “wet macular degeneration,” “neovascularization,” and “glaucoma.” From the 8 prompts extracted from the Chinese guideline, 2 were excluded: “How should anti-VEGF agents be selected for the treatment of neovascular AMD?” and “What is the optimal anti-VEGF treatment regimen for neovascular AMD?” All 10 topic prompts from the American Academy of Ophthalmology Preferred Practice Pattern were retained, resulting in the final set of 30 prompts.

### LLMs and Query Process

Six publicly available, general-purpose conversational LLMs were evaluated: ChatGPT-5.1-auto (OpenAI; August 7, 2025), DeepSeek-V3.2-Exp, displayed in the public interface as DeepSeek-v3.2 (DeepSeek; September 25, 2025), Gemini-2.5-Flash-Thinking (Google; June 17, 2025), Grok 4 (xAI; July 9, 2025), Claude Sonnet 4.5 (Anthropic; September 29, 2025), and Qwen3-Max (Alibaba Cloud; September 24, 2025). These dates were used to document the public model versions or modes available during the data collection period and do not imply access to fixed back-end model-weight snapshots. These models were selected because they were broadly accessible during the study period, represented major international and Chinese AI developers, were available as identifiable model versions or modes, and could be evaluated under the same zero-shot, prompt-only procedure. Dedicated retrieval-augmented answer engines designed primarily around live search and source ranking, such as Perplexity, were not included. However, this exclusion did not mean that all evaluated public web interfaces functioned as pure stand-alone base models. The study evaluated each model as encountered by a typical public user under default interface settings. During review of the raw outputs, ChatGPT-5.1-auto and Qwen3-Max displayed retrieval- or citation-related artifacts in at least some default interface responses, including source links or Urchin Tracking Module (UTM)–tracked links and bracketed web citations. These features were not manually activated, disabled, standardized, or selectively removed. Therefore, the results should be interpreted as a comparison of default public interface responses rather than a controlled comparison of nonretrieval stand-alone model capabilities. All queries were conducted between October 10 and November 25, 2025, through the official publicly accessible web interfaces of the 6 evaluated models. No API calls, automated scripts, browser extensions, plug-ins, or programmatic access methods were used. Each finalized English-language prompt was manually entered by the authors into each model interface, and the corresponding response was manually extracted and recorded for analysis. All Google Trends searches and LLM queries were conducted in private or incognito browsing mode after signing out of Google and other personal accounts. Browser cache and cookies were cleared before each session to minimize local browser session effects. All prompts and model outputs included in the analysis were in English. Each prompt was entered in a separate new session, without prior conversational history, additional context, custom instructions, user-specified system prompts, repeated prompt optimization, regeneration, or selective response replacement. Default public user interface settings were used. Accordingly, API-level generation parameters, including temperature, top-p, maximum token length, and frequency or presence penalties, were not displayed to or controllable by the investigators and were not manually set. A single response from each model for each prompt was collected for analysis.

### Response Preprocessing and Blinding

Before scoring, all model responses were transferred from the original model-specific files into a standardized scoring template and assigned anonymized response codes. Model names, file names, interface labels, logos, time stamps, ordering information, and any explicit self-identifying boilerplate text indicating the model name or developer were manually removed or redacted before rater review. This redaction was limited strictly to model-identifying information and did not alter any substantive clinical content, wording, recommendations, references, uncertainty statements, or safety-related statements in the responses. However, URL-level tracking parameters embedded within reference links, including UTM parameters, were not systematically scrubbed before scoring because references were otherwise preserved to avoid altering attribution-related content. Therefore, although explicit model names, interface labels, and self-identifying boilerplate were removed, some reference URLs may have retained model-identifying tracking strings. The 2 ophthalmologist raters received only anonymized prompt response pairs and were not informed of the model source. When discrepancies occurred, a third ophthalmologist (CM) adjudicated the score using the same anonymized materials.

### Outcome Measures

#### Reliability Assessment

Two board-certified ophthalmologists (ZL and X.M) with over 10 years of clinical experience independently evaluated the anonymized and preprocessed responses using 4 established tools while remaining blinded to the model sources. In case of disagreement between the 2 raters, a third ophthalmologist of equivalent qualification served as the final adjudicator.

#### DISCERN

DISCERN was used to assess the quality of information regarding “treatment choices” in patient education materials [19]. It consists of 16 items divided into 3 sections: reliability of information (items 1‐8), specific quality of information on treatment choices (items 9‐15), and an overall rating (item 16). Each item is scored from 1 to 5, with a total score range of 16‐80. Higher scores indicate better overall quality. For this study, scores were defined as follows: 16‐26: “very poor,” 27‐38: “poor,” 39‐50: “fair,” 51‐62: “good,” and 63‐80: “excellent.” [20]

#### Ensuring Quality Information for Patients

EQIP was used to assess the overall quality of health education materials regarding information sources, accuracy, comprehensibility, and presentation format [21]. It consists of 20 items. Each item is answered “Yes/No,” with “Yes” scoring 1 point and “No” scoring 0 points. The final score is the percentage of “Yes” items out of the total (ie, proportion of “Yes” × 100). Higher scores indicate better material quality [22].

#### Global Quality Scale

GQS is a single-item, 5-point Likert scale for subjective overall evaluation of health education materials: 1 point for “Very poor,” 2 for “Poor,” 3 for “Fair,” 4 for “Good,” and 5 for “Excellent.”

#### JAMA Benchmark Criteria

JAMA criteria focus on the standardization and transparency of online health information, including 4 items: authorship, attribution of sources and references, disclosure of conflicts of interest (or sponsorship or advertising), and currency. Each item is scored “Yes/No,” with “Yes” scoring 1 point and “No” scoring 0 points, for a total score of 0‐4. Higher scores indicate better performance in information transparency and standardization [23]. In this study, the JAMA benchmark was applied uniformly across all model outputs as a structured proxy for visible transparency and verifiability features within the response text, rather than as a direct measure of factual accuracy, clinical safety, true human authorship, or actual financial conflicts of interest. Because citation display may be an interface-level feature rather than an intrinsic property of the underlying model, JAMA benchmark scores in this study should be interpreted as visible transparency under default public interface conditions.

### Hallucination Assessment Scope

For transparency, hallucination was operationally defined as fabricated clinical facts, guideline-inconsistent recommendations, unsupported certainty regarding diagnosis or treatment, or nonverifiable references presented as evidence. However, hallucination was not prespecified as a stand-alone analytic end point in this study. During clinical evaluation, raters assessed the informational reliability, transparency indicators, overall quality, and readability of model responses using DISCERN, EQIP, GQS, JAMA benchmark criteria, and readability formulas. Although unsupported or misleading content could influence these quality scores, the raters did not perform claim-level fact checking, assign binary hallucination labels, or record model-level hallucination frequencies. Therefore, hallucination frequency was not reported as an independent outcome.

### Readability Assessment

Because all prompts and model outputs analyzed in this study were in English, English-language readability formulas were used. No Chinese-language prompts, untranslated Chinese-language prompts, or Chinese-language model outputs were included in the readability assessment.

Responses were analyzed using six standard readability formulas calculated via an online tool (readabilityformulas.com):

1. Automated Readability Index (ARI) [24]:


$$4.71\left(\frac{\text{Characters}}{\text{Words}}\right)+0.5\left(\frac{\text{Words}}{\text{Sentences}}\right)-21.43$$

2. Flesch Reading Ease Score (FRES) [25]:


$$206.835-1.015\left(\frac{\text{Words}}{\text{Sentences}}\right)-84.6\left(\frac{\text{Syllables}}{\text{Words}}\right)$$

3. Gunning Fog Index (GFI) [25]:


$$0.4\left[\left(\frac{\text{Words}}{\text{Sentences}}\right)+100\left(\frac{\text{Complex words}}{\text{Words}}\right)\right]$$

4. Flesch-Kincaid Grade Level (FKGL) [25]:


$$0.39\left(\frac{\text{Words}}{\text{Sentences}}\right)+11.8\left(\frac{\text{Syllables}}{\text{Words}}\right)-15.59$$

5. Coleman-Liau Index (CL) [25]:


$$\begin{array}{r} 5.89\left(\frac{\text{Characters}}{\text{Words}}\right)\\ \;-0.3\left(\frac{\text{Sentences}}{\text{Words}}\right)-15.8 \end{array}$$

6. Simple Measure of Gobbledygook (SMOG) [25]:


$$14.430\times \sqrt{\text{Polysyllables }\times \frac{30}{sentences}}+3.1291$$

The readability scores were compared and analyzed with the sixth-grade level of readability recommended by the American Medical Association and the National Institutes of Health [26]. This threshold was considered appropriate for the education of patients with AMD because it has been used in prior studies of AMD-specific and geriatric patient education materials, and because AMD predominantly affects older adults, for whom central visual impairment may further increase the practical burden of reading and processing complex written health information [5,27]. Following prior readability studies, including the referenced *PeerJ* article, the accepted readability level was defined as ≥80.0 for the FRES and ≤6 for the other 5 grade-level formulas. For interpretation, lower FRES values indicate more difficult reading, whereas higher ARI, GFI, FKGL, CL, and SMOG values indicate greater reading difficulty; therefore, FRES values below 80.0 or grade-level formula scores above 6 were interpreted as exceeding the recommended sixth-grade readability level.

### Statistical Analysis

#### Overview

All statistical analyses were performed using IBM SPSS Statistics (version 29.0; IBM Corp) and R (version 4.3.2; R Core Team). First, the mean and standard deviation for each of the 6 models across all 30 prompts were calculated. Interrater agreement between the 2 independent assessors on the 4 reliability measures (DISCERN, EQIP, GQS, and JAMA benchmark criteria) was calculated using Cohen κ. Cohen κ values and corresponding 95% CIs were calculated from the paired preadjudication ratings of the 2 independent assessors. Agreement was categorized as follows: κ≤0, no agreement; 0.01‐0.20, slight; 0.21‐0.40, fair; 0.41‐0.60, moderate; 0.61‐0.80, substantial; and 0.81‐1.00, almost perfect agreement.

#### Reliability Analysis

Cohen κ was used to assess interrater agreement for the 4 reliability tools. Descriptive statistics (mean, SD) were calculated for each model across all 30 prompts for each measure. The Friedman test was used to examine overall differences among the 6 models for each measure, followed by post hoc pairwise comparisons with Holm-Bonferroni adjustment for multiple comparisons when significant. A *P* value of <.05 was set as the threshold for statistical significance. Composite reliability and readability *z* scores were calculated as described in the Statistical Analysis subsection and were used to generate the composite visualization.

#### Readability Analysis

Readability scores were calculated for each model’s responses using the established readability metrics. Given the repeated-measures design, in which each of the 30 standardized AMD prompts was answered by all 6 models, the Friedman test was used to compare differences in readability scores among the 6 models. When the overall Friedman test was significant, post hoc pairwise comparisons were performed with Holm-Bonferroni adjustment. To assess whether the generated text met the sixth-grade reading level, the Wilcoxon signed-rank test was used to compare readability scores against the standardized sixth-grade threshold. *P* values were calculated for all tests, with *P*<.05 considered statistically significant.

**Figure 1.**
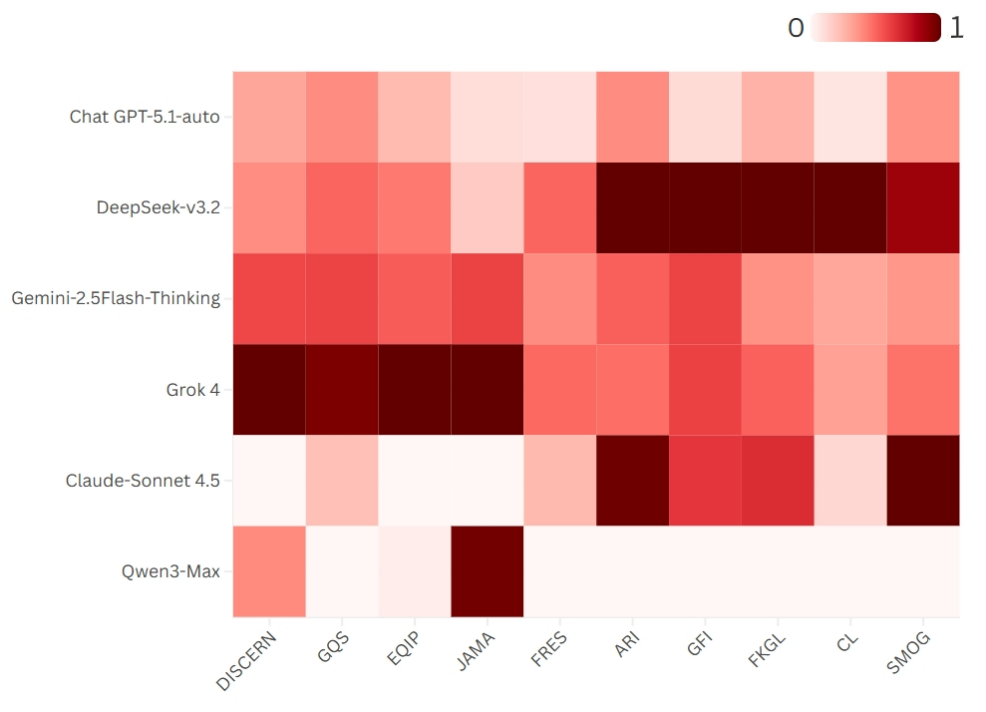
Normalized performance heatmap of 6 large language models across reliability (DISCERN, EQIP, GQS, and JAMA benchmark criteria) and readability (ARI, FRES, GFI, FKGL, CL, and SMOG) metrics. Darker shades indicate better relative performance per metric (columns normalized independently). ARI: Automated Readability Index; CL: Coleman-Liau Index; EQIP: Ensuring Quality Information for Patients; FKGL: Flesch-Kincaid Grade Level; FRES: Flesch Reading Ease Score; GFI: Gunning Fog Index; GQS: Global Quality Scale; JAMA: Journal of the American Medical Association benchmark criteria; SMOG: Simple Measure of Gobbledygook.

Regarding Figure 1, average scores for each model were normalized by indicator using minimum-maximum normalization, resulting in scores ranging from 0 to 1.

For “higher-is-better” indicators, the following equation was used:


$$S_{m,k}=\frac{{\bar{x}}_{m,k}-\min_{k}}{\max_{k}-\min_{k}}$$

For “lower-is-better” readability grade-level indicators, the following equation was used:


$$S_{m,k}=\frac{\max_{k}-{\bar{x}}_{m,k}}{\max_{k}-\min_{k}}$$

**Figure 2.**
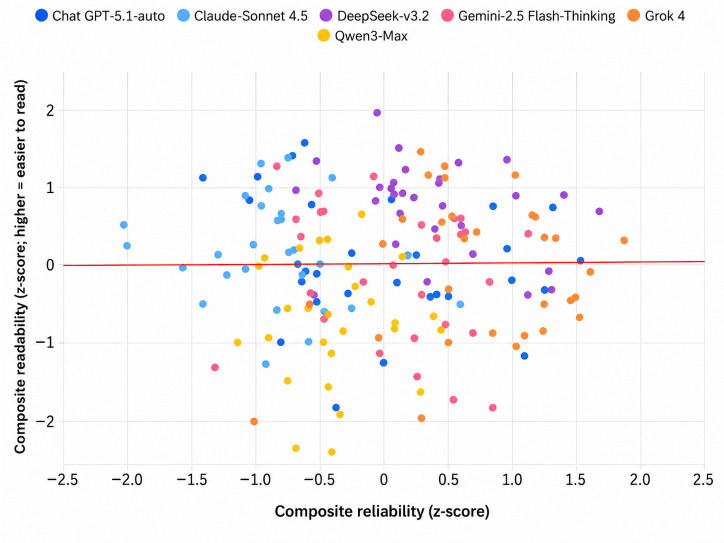
Scatterplot of composite reliability *z* scores versus composite readability *z* scores for each model response (N=180). The red line indicates the overall trend across all responses.

Regarding Figure 2, all 180 observations were Z-standardized:


$$Z_{i,k}=\frac{x_{i,k}-\mu_{k}}{\sigma_{k}}$$

For “lower-is-better” readability grade-level indicators, the sign was inverted:


$$Z_{i,k}^{*}=-Z_{i,k}$$

Composite reliability and readability were calculated as unweighted averages:


$$R_{i}=\frac{1}{4}\sum Z_{i,k}^{*},\;Q_{i}=\frac{1}{6}\sum Z_{i,k}^{*}$$

**Figure 3.**
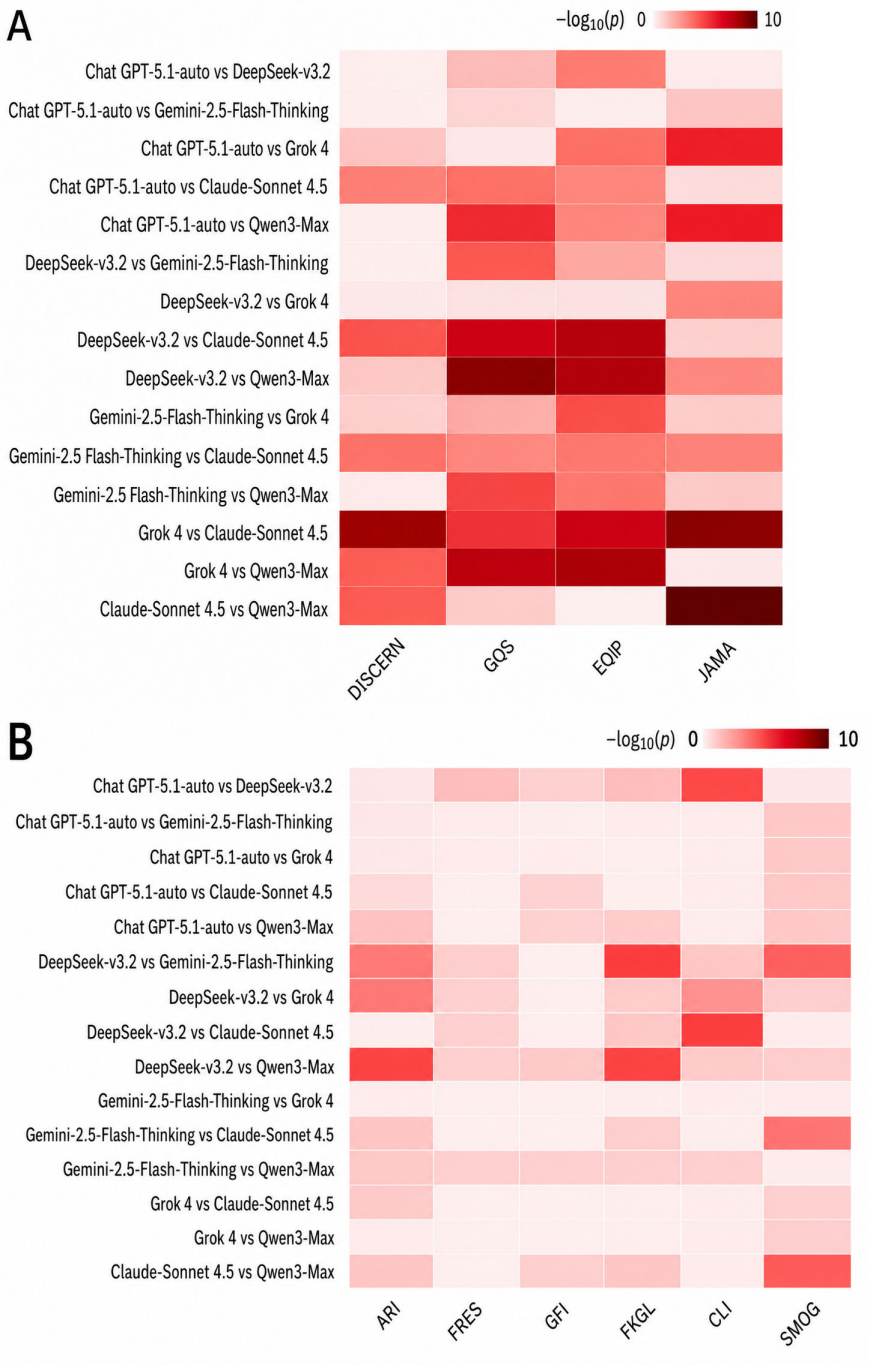
Heatmaps of pairwise model differences across reliability and readability metrics. (A) Pairwise differences for reliability metrics, including DISCERN, GQS, EQIP, and JAMA benchmark criteria. (B) Pairwise differences for readability metrics, including ARI, FRES, GFI, FKGL, CL, and SMOG. Values represent −log10 (Holm-adjusted *P* values) from post hoc paired Wilcoxon signed-rank comparisons following significant Friedman tests. Darker cells denote more statistically significant between-model differences. ARI: Automated Readability Index; CL: Coleman-Liau Index; EQIP: Ensuring Quality Information for Patients; FKGL: Flesch-Kincaid Grade Level; FRES: Flesch Reading Ease Score; GFI: Gunning Fog Index; GQS: Global Quality Scale; JAMA: Journal of the American Medical Association benchmark criteria; SMOG: Simple Measure of Gobbledygook.

Regarding Figure 3, the Friedman test was used to compare overall differences among the 6 models for each indicator:


$$X_{F}^{2}=\frac{12}{nk(k+1)}\sum R_{j}^{2}-3n(k+1)$$

If significant, pairwise comparisons were conducted. The standardized test statistic was:


$$Z=\sqrt{\frac{\left|\bar{r_{a}}-\bar{r_{b}}\right|}{\frac{k(k+1)}{6n}}}$$

The Holm-Bonferroni correction was applied. The heatmap displays $-\log_{\text{10}}\text{(}{\text{p}}_{\text{Holm}}\text{)}$.

### Ethical Considerations

This study did not involve human participants, direct interaction with individuals, identifiable private information, or individual-level patient data. The analysis was based on publicly available search-trend information, published clinical guidelines and recommendations, and textual responses generated by publicly accessible LLMs. Therefore, formal ethics review was not required, informed consent was not applicable, and no ethics approval number was assigned. No personal identifiers, private user information, or patient records were collected or analyzed. No compensation was provided because no participants were recruited. The manuscript and supplementary materials do not contain images, screenshots, or other content that could identify individual participants or users.

## Results

### Overview

A total of 180 responses (30 prompts × 6 models) were analyzed. Interrater agreement for all 4 reliability instruments was substantial to almost perfect, with κ values of 0.72 for DISCERN (95% CI 0.635‐0.802), 0.80 for EQIP (95% CI 0.717‐0.875), 0.92 for GQS (95% CI 0.864‐0.959), and 0.97 for JAMA benchmark criteria (95% CI 0.942‐0.988). These findings indicated substantial to almost perfect preadjudication agreement between the 2 independent ophthalmologist assessors (ZL and XM); all scoring discrepancies were subsequently resolved by a third ophthalmologist (CM) of equivalent qualification, who served as the final adjudicator using the same anonymized materials.

### Reliability Scores

**Figure 4.**
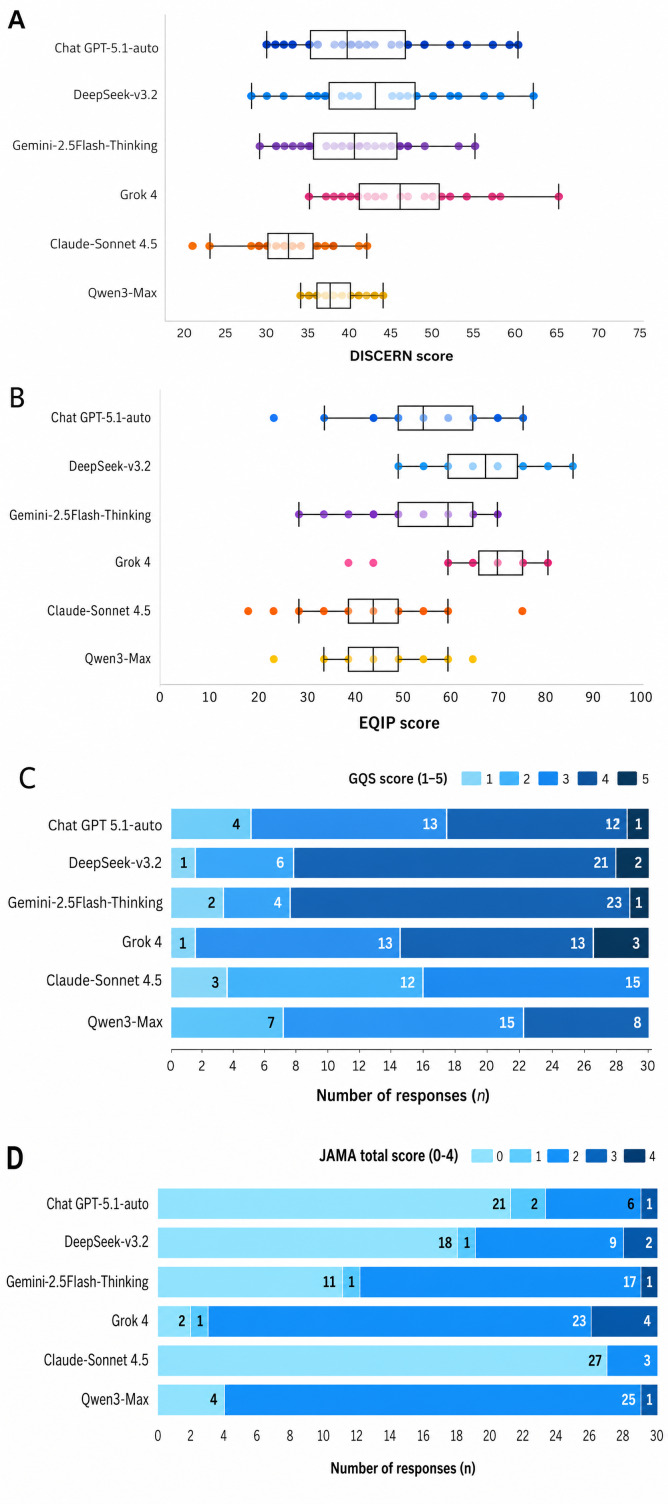
Distribution of reliability scores across 6 large language models (violin plots). (A) DISCERN scores, (B) EQIP scores, (C) GQS scores, and (D) JAMA benchmark scores. EQIP: Ensuring Quality Information for Patients; GQS: Global Quality Scale; JAMA: Journal of the American Medical Association benchmark criteria.

Substantial between-model variation was observed across all reliability-related metrics. As shown in Table 1, Grok 4 achieved the highest mean DISCERN score (mean 46.40, SD 7.43), followed by DeepSeek-v3.2 (mean 43.33, SD 8.20). A similar pattern was observed for EQIP, where Grok 4 (mean 74.33, SD 9.07) and DeepSeek-v3.2 (mean 71.50, SD 9.11) outperformed the remaining models. For the JAMA benchmark criteria, Grok 4 generated the highest mean score (mean 1.97, SD 0.67), but Qwen3-Max showed a similarly high JAMA score (mean 1.90, SD 0.40), indicating comparatively strong visible transparency despite lower scores on DISCERN, GQS, and EQIP. Claude-Sonnet 4.5 showed the lowest overall reliability-related profile across the evaluated metrics, whereas Qwen3-Max showed lower non-JAMA reliability scores but relatively strong JAMA benchmark performance. These distributional differences are illustrated by the violin plots in Figure 4A-D, which present DISCERN, EQIP, GQS, and JAMA benchmark score distributions, respectively.

**Table 1.** Reliability scores (N=30 prompts).

	DISCERN	GQS^a^	EQIP^b^	JAMA^c^
ChatGPT-5.1-auto, mean (SD)	41.13 (8.89)	3.33 (0.76)	61.17 (12.08)	0.57 (0.94)
DeepSeek-v3.2, mean (SD)	43.33 (8.20)	3.70 (0.65)	71.50 (9.11)	0.83 (1.09)
Gemini-2.5Flash-Thinking, mean (SD)	40.73 (6.59)	3.03 (0.49)	62.33 (10.73)	1.27 (1.01)
Grok 4, mean (SD)	46.40 (7.43)	3.60 (0.72)	74.33 (9.07)	1.97 (0.67)
Claude-Sonnet 4.5, mean (SD)	32.67 (4.62)	2.40 (0.67)	49.00 (10.70)	0.20 (0.61)
Qwen3-Max, mean (SD)	38.07 (3.06)	2.03 (0.72)	50.00 (9.19)	1.90 (0.40)
*P* value	<.001	<.001	<.001	<.001

a GQS: Global Quality Scale.

b EQIP: Ensuring Quality Information for Patients.

c JAMA; Journal of the American Medical Association benchmark criteria.

### Readability Scores

Readability assessments demonstrated that all models produced text considerably exceeding the recommended sixth-grade reading level (Table 2). This interpretation was based on the predefined cutoffs: all mean FRES values were below the accepted threshold of 80.0, and all mean grade-level formula scores were above 6, indicating text harder than the recommended sixth-grade level.

**Table 2.** Readability metrics (N=30 prompts).

	ARI^a^	FRES^b^	GFI^c^	FKGL^d^	CL^e^	SMOG^f^
ChatGPT-5.1-auto, mean (SD)	13.16 (3.95)	27.50 (14.29)	14.87 (3.25)	12.47 (3.49)	15.20 (2.62)	10.49 (3.24)
DeepSeek-v3.2, mean (SD)	11.90 (2.14)	48.23 (9.16)	12.26 (1.81)	9.95 (1.87)	11.91 (1.75)	9.98 (1.50)
Gemini-2.5 Flash-Thinking, mean (SD)	14.67 (3.20)	32.60 (13.78)	13.62 (2.84)	13.34 (2.85)	14.59 (1.85)	12.07 (2.38)
Grok 4, mean (SD)	14.89 (3.38)	35.17 (17.21)	13.59 (2.37)	12.61 (3.28)	14.53 (2.61)	11.63 (2.45)
Claude-Sonnet 4.5, mean (SD)	12.06 (2.04)	28.87 (13.06)	13.47 (2.40)	11.70 (1.99)	15.28 (1.97)	9.40 (1.59)
Qwen3-Max, mean (SD)	17.59 (3.23)	22.60 (14.23)	15.65 (2.62)	15.37 (2.85)	15.78 (2.43)	13.57 (2.49)
Sixth-grade level score	6	80	6	6	6	6
Overall *P* value, Friedman test	<.001	<.001	<.001	<.001	<.001	<.001

a ARI: Automated Readability Index.

b FRES: Flesch Reading Ease Score.

c GFI: Gunning Fog Index.

d FKGL: Flesch-Kincaid Grade Level.

e CL: Coleman-Liau Index.

f SMOG: Simple Measure of Gobbledygook.

**Figure 5.**
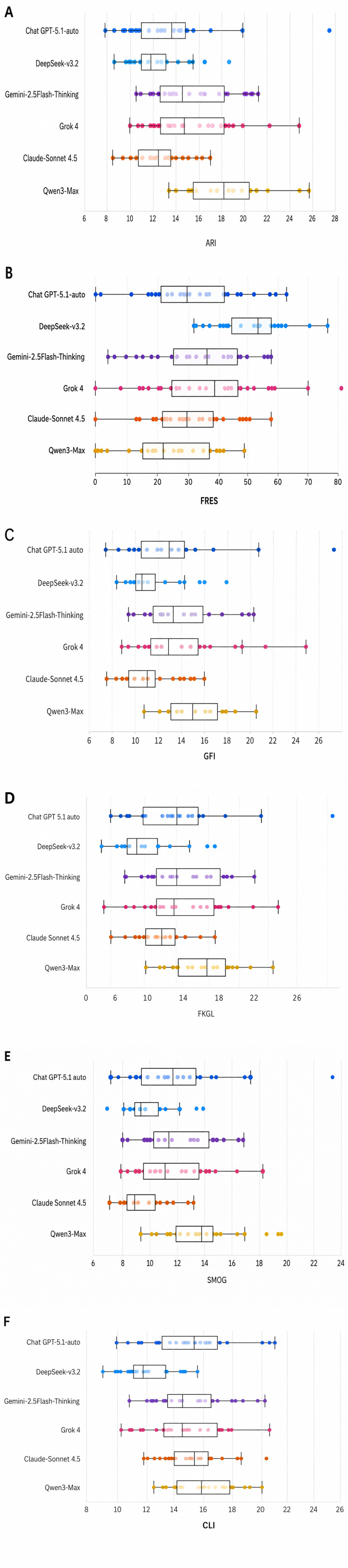
Distribution of readability scores across 6 large language models (violin plots). (A) ARI, (B) FRES, (C) GFI, (D) FKGL, (E) SMOG, and (F) CL. ARI: Automated Readability Index; CL: Coleman-Liau Index; FKGL: Flesch-Kincaid Grade Level; FRES: Flesch Reading Ease Score; GFI: Gunning Fog Index; SMOG: Simple Measure of Gobbledygook.

DeepSeek-v3.2 showed the most favorable overall readability profile, yielding the highest FRES score (mean 48.23, SD 9.16) and the lowest scores for ARI (mean 11.90, SD 2.14), GFI (mean 12.26, SD 1.81), FKGL (mean 9.95, SD 1.87), and CL (mean 11.91, SD 1.75). However, Claude-Sonnet 4.5 achieved the lowest SMOG score (mean 9.40, SD 1.59), followed by DeepSeek-v3.2 (mean 9.98, SD 1.50). In contrast, Gemini-2.5 Flash-Thinking, Grok 4, and Qwen3-Max produced text requiring substantially higher reading proficiency, with Qwen3-Max reaching the highest FKGL (mean 15.37, SD 2.85) and SMOG (mean 13.57, SD 2.49) levels. Violin plots in Figure 5 further demonstrate clear intermodel divergence in readability distributions. Wilcoxon signed-rank tests comparing readability scores with the prespecified sixth-grade thresholds confirmed that none of the 6 models met the recommended readability level. For ARI, GFI, FKGL, CL, and SMOG, values were significantly above the threshold of 6.0 across all models, whereas FRES values were significantly below the threshold of 80.0.

### Integrated Performance Visualization

After standardization, the cross-metric performance heatmap (Figure 1) revealed distinct model profiles. Grok 4 performed strongly across reliability-related metrics, particularly DISCERN, EQIP, and JAMA benchmark criteria, but this reliability advantage was not accompanied by favorable readability. Its responses showed mixed and generally less favorable readability than those of DeepSeek-v3.2. DeepSeek-v3.2 showed the most balanced performance profile, combining comparatively strong reliability with the most favorable overall readability. Consistent with Table 2, the composite *z* score scatterplots (Figure 2) showed that Grok 4 responses were distributed toward the high-reliability side but extended substantially into lower-readability regions, whereas DeepSeek-v3.2 occupied the more favorable reliability-readability region. Other models showed less favorable profiles across either reliability or readability dimensions.

### Overall Between-Model Differences

Friedman tests confirmed significant between-model differences across all reliability and readability metrics (*χ*²_5_ range, 43.859‐93.887; Kendall W range, 0.292‐0.626; all *P*<.001) (Table 3). Reliability indices showed the strongest effect sizes, particularly for GQS (*χ*²_5_=93.887; W=0.626) and EQIP (*χ*²_5_=82.132; W=0.548). Readability metrics also demonstrated robust intermodel variability, with the largest differences seen in SMOG (*χ*²_5_=73.105; W=0.487) and FKGL (*χ*²_5_=70.367; W=0.469).

**Table 3.** Overall between-model differences (Friedman test; within-prompt paired, N=30; k=6).

	Friedman χ² (*df*)	Kendall W	*P* value (raw)	*P* value (Holm)
DISCERN	69.845 (5)	0.466	<.001	<.001
GQS^a^	93.887 (5)	0.626	<.001	<.001
EQIP^b^	82.132 (5)	0.548	<.001	<.001
JAMA^c^	72.669 (5)	0.484	<.001	<.001
ARI^d^	65.524 (5)	0.437	<.001	<.001
FRES^e^	68.5 (5)	0.457	<.001	<.001
GFI^f^	43.859 (5)	0.292	<.001	<.001
FKGL^g^	70.367 (5)	0.469	<.001	<.001
CL^h^	58.381 (5)	0.389	<.001	<.001
SMOG^i^	73.105 (5)	0.487	<.001	<.001

a GQS: Global Quality Scale.

b EQIP: Ensuring Quality Information for Patients.

c JAMA: Journal of the American Medical Association benchmark criteria.

d ARI: Automated Readability Index.

e FRES: Flesch Reading Ease Score.

f GFI: Gunning Fog Index.

g FKGL: Flesch-Kincaid Grade Level.

h CL: Coleman-Liau Index.

i SMOG: Simple Measure of Gobbledygook.

### Pairwise Comparisons

Holm-adjusted post hoc pairwise comparisons for reliability metrics are reported in Table 4, whereas corresponding pairwise comparisons for readability metrics are reported in Table 5. For reliability metrics, Grok 4 significantly outperformed Claude-Sonnet 4.5 across all 4 reliability-related metrics and outperformed Qwen3-Max on DISCERN, GQS, and EQIP but not on JAMA benchmark criteria. DeepSeek-v3.2 also showed significantly higher EQIP scores than several comparator models. For readability metrics, DeepSeek-v3.2 generated significantly easier-to-read responses than Gemini-2.5 Flash-Thinking, Grok 4, and Qwen3-Max across multiple readability indices, including ARI, FRES, FKGL, CL, and SMOG. These pairwise differences are visualized in Figure 3A for reliability metrics and Figure 3B for readability metrics. The corresponding Wilcoxon signed-rank statistics and exact 2-sided *P* values are reported in Table 6.

**Table 4.** Holm-adjusted post hoc pairwise comparison *P* values for reliability metrics.

Pair	DISCERN	GQS^a^	EQIP^b^	JAMA^c^
ChatGPT-5.1-auto vs DeepSeek-v3.2	.24	.02	.001	.35
ChatGPT-5.1-auto vs Gemini-2.5Flash-Thinking	.85	.06	.69	.009
ChatGPT-5.1-auto vs Grok 4	.01	.22	<.001	<.001
ChatGPT-5.1-auto vs Claude-Sonnet 4.5	<.001	<.001	<.001	.06
ChatGPT-5.1-auto vs Qwen3-Max	.46	<.001	<.001	<.001
DeepSeek-v3.2 vs Gemini-2.5Flash-Thinking	.22	<.001	.002	.12
DeepSeek-v3.2 vs Grok 4	.11	.26	.12	<.001
DeepSeek-v3.2 vs Claude-Sonnet 4.5	<.001	<.001	<.001	.008
DeepSeek-v3.2 vs Qwen3-Max	.004	<.001	<.001	<.001
Gemini-2.5Flash-Thinking vs Grok 4	.005	.001	<.001	.004
Gemini-2.5Flash-Thinking vs Claude-Sonnet 4.5	<.001	<.001	<.001	<.001
Gemini-2.5Flash-Thinking vs Qwen3-Max	.08	<.001	<.001	.01
Grok 4 vs Claude-Sonnet 4.5	<.001	<.001	<.001	<.001
Grok 4 vs Qwen3-Max	<.001	<.001	<.001	.30
Claude-Sonnet 4.5 vs Qwen3-Max	<.001	.047	.81	<.001

a GQS: Global Quality Scale.

b EQIP: Ensuring Quality Information for Patients.

c JAMA: Journal of the American Medical Association benchmark criteria.

**Table 5.** Holm-adjusted post hoc pairwise comparison *P* values for readability metrics.

Pair	ARI^a^	FRES^b^	GFI^c^	FKGL^d^	CL^e^	SMOG^f^
ChatGPT-5.1-auto vs DeepSeek-v3.2	≥.99	<.001	.001	.006	<.001	.89
ChatGPT-5.1-auto vs Gemini-2.5Flash-Thinking	.10	.46	.25	.37	≥.99	.01
ChatGPT-5.1-auto vs Grok 4	.046	.11	.10	.42	≥.99	.03
ChatGPT-5.1-auto vs Claude-Sonnet 4.5	.58	≥.99	.07	.42	≥.99	.29
ChatGPT-5.1-auto vs Qwen3-Max	<.001	.19	.17	.002	.77	<.001
DeepSeek-v3.2 vs Gemini-2.5Flash-Thinking	<.001	<.001	.01	<.001	<.001	<.001
DeepSeek-v3.2 vs Grok 4	<.001	.002	.009	<.001	<.001	<.001
DeepSeek-v3.2 vs Claude-Sonnet 4.5	≥.99	<.001	.0498	.002	<.001	.29
DeepSeek-v3.2 vs Qwen3-Max	<.001	<.001	<.001	<.001	<.001	<.001
Gemini-2.5Flash-Thinking vs Grok 4	≥.99	≥.99	≥.99	.29	≥.99	.29
Gemini-2.5Flash-Thinking vs Claude-Sonnet 4.5	<.001	.46	≥.99	.01	.20	<.001
Gemini-2.5Flash-Thinking vs Qwen3-Max	.003	.02	.004	.04	.08	.04
Grok 4 vs Claude-Sonnet 4.5	.001	.46	≥.99	.42	≥.99	<.001
Grok 4 vs Qwen3-Max	.004	.003	.003	.003	.13	.01
Claude-Sonnet 4.5 vs Qwen3-Max	<.001	.03	.001	<.001	≥.99	<.001

a ARI: Automated Readability Index.

b FRES: Flesch Reading Ease Score.

c GFI: Gunning Fog Index.

d FKGL: Flesch-Kincaid Grade Level.

e CL: Coleman-Liau Index.

f SMOG: Simple Measure of Gobbledygook.

**Table 6.** Wilcoxon signed-rank tests comparing readability scores with sixth-grade readability thresholds^a^.

Model	Metric	Threshold	Mean (SD)	Median	W	Exact *P* value	Direction
ChatGPT-5.1-auto	ARI^b^	6	13.16 (3.95)	12.97	0	1.86×10⁻⁹	Above threshold
ChatGPT-5.1-auto	FRES^c^	80	27.50 (14.29)	27.00	0	1.86×10⁻⁹	Below threshold
ChatGPT-5.1-auto	GFI^d^	6	14.87 (3.25)	15.00	0	1.86×10⁻⁹	Above threshold
ChatGPT-5.1-auto	FKGL^e^	6	12.47 (3.49)	12.13	0	1.86×10⁻⁹	Above threshold
ChatGPT-5.1-auto	CL^f^	6	15.20 (2.62)	15.48	0	1.86×10⁻⁹	Above threshold
ChatGPT-5.1-auto	SMOG^g^	6	10.49 (3.24)	9.77	0	1.86×10⁻⁹	Above threshold
DeepSeek-v3.2	ARI	6	11.90 (2.14)	11.38	0	1.86×10⁻⁹	Above threshold
DeepSeek-v3.2	FRES	80	48.23 (9.16)	50.00	0	1.86×10⁻⁹	Below threshold
DeepSeek-v3.2	GFI	6	12.26 (1.81)	12.10	0	1.86×10⁻⁹	Above threshold
DeepSeek-v3.2	FKGL	6	9.95 (1.87)	9.44	0	1.86×10⁻⁹	Above threshold
DeepSeek-v3.2	CL	6	11.91 (1.75)	11.52	0	1.86×10⁻⁹	Above threshold
DeepSeek-v3.2	SMOG	6	9.98 (1.50)	9.65	0	1.86×10⁻⁹	Above threshold
Gemini-2.5Flash-Thinking	ARI	6	14.67 (3.20)	13.82	0	1.86×10⁻⁹	Above threshold
Gemini-2.5Flash-Thinking	FRES	80	32.60 (13.78)	34.50	0	1.86×10⁻⁹	Below threshold
Gemini-2.5Flash-Thinking	GFI	6	13.62 (2.84)	13.70	0	1.86×10⁻⁹	Above threshold
Gemini-2.5Flash-Thinking	FKGL	6	13.34 (2.85)	12.27	0	1.86×10⁻⁹	Above threshold
Gemini-2.5Flash-Thinking	CL	6	14.59 (1.85)	14.29	0	1.86×10⁻⁹	Above threshold
Gemini-2.5Flash-Thinking	SMOG	6	12.07 (2.38)	11.23	0	1.86×10⁻⁹	Above threshold
Grok 4	ARI	6	14.89 (3.38)	13.97	0	1.86×10⁻⁹	Above threshold
Grok 4	FRES	80	35.17 (17.21)	37.00	0	1.86×10⁻⁹	Below threshold
Grok 4	GFI	6	13.59 (2.37)	13.35	0	1.86×10⁻⁹	Above threshold
Grok 4	FKGL	6	12.61 (3.28)	11.52	0	1.86×10⁻⁹	Above threshold
Grok 4	CL	6	14.53 (2.61)	14.48	0	1.86×10⁻⁹	Above threshold
Grok 4	SMOG	6	11.63 (2.45)	11.04	0	1.86×10⁻⁹	Above threshold
Claude-Sonnet 4.5	ARI	6	12.06 (2.04)	12.03	0	1.86×10⁻⁹	Above threshold
Claude-Sonnet 4.5	FRES	80	28.87 (13.06)	29.00	0	1.86×10⁻⁹	Below threshold
Claude-Sonnet 4.5	GFI	6	13.47 (2.40)	13.45	0	1.86×10⁻⁹	Above threshold
Claude-Sonnet 4.5	FKGL	6	11.70 (1.99)	11.87	0	1.86×10⁻⁹	Above threshold
Claude-Sonnet 4.5	CL	6	15.28 (1.97)	15.32	0	1.86×10⁻⁹	Above threshold
Claude-Sonnet 4.5	SMOG	6	9.40 (1.59)	8.87	0	1.86×10⁻⁹	Above threshold
Qwen3-Max	ARI	6	17.59 (3.23)	17.39	0	1.86×10⁻⁹	Above threshold
Qwen3-Max	FRES	80	22.60 (14.23)	22.00	0	1.86×10⁻⁹	Below threshold
Qwen3-Max	GFI	6	15.65 (2.62)	15.50	0	1.86×10⁻⁹	Above threshold
Qwen3-Max	FKGL	6	15.37 (2.85)	15.38	0	1.86×10⁻⁹	Above threshold
Qwen3-Max	CL	6	15.78 (2.43)	15.75	0	1.86×10⁻⁹	Above threshold
Qwen3-Max	SMOG	6	13.57 (2.49)	13.59	0	1.86×10⁻⁹	Above threshold

a For ARI, GFI, FKGL, CL, and SMOG, the sixth-grade threshold was defined as ≤6; values above 6 indicate text harder than the recommended level. For FRES, the threshold was defined as ≥80; values below 80 indicate text harder than the recommended level. Exact 2-sided *P* values are reported. No zero differences occurred in these signed-rank comparisons; therefore, the full sample of 30 prompt-level observations was retained for each model-metric comparison.

b ARI: Automated Readability Index.

c FRES: Flesch Reading Ease Score.

d GFI: Gunning Fog Index.

e FKGL: Flesch-Kincaid Grade Level.

f CL: Coleman-Liau Index.

g SMOG: Simple Measure of Gobbledygook.

## Discussion

### Principal Findings

In this comparative evaluation of 6 publicly accessible LLMs for the education of patients with AMD, we found substantial model-dependent differences in informational reliability, transparency indicators, overall quality, and readability. No model met the recommended sixth-grade readability benchmark under zero-shot, single-turn prompting conditions [26]. These findings suggest a persistent reliability-readability gap in current LLM-generated education materials of patients with AMD. This gap is clinically relevant because the education of patients with AMD requires information that is not only reliable and transparent but also understandable for older adults who may face visual and health literacy barriers.

### Comparison With Prior Work

Building on prior studies reporting that AI-generated patient education materials often remain above-recommended readability levels despite apparent coherence and informativeness, our findings show that higher informational reliability does not necessarily correspond to better readability [4,28]. This pattern is substantiated by the distinct model profiles shown in Figures 1, 4A-D, and 5A-F, and Tables 1 and 2. This divergence appears rooted in underlying design philosophies. Grok 4, which achieved the highest mean scores in DISCERN (mean 46.40, SD 7.43) and EQIP (mean 74.33, SD 9.07) (Table 1), concurrently produced text with among the highest grade–level indices (eg, ARI: mean 14.89, SD 3.38) (Table 2). In Figure 2, Grok 4 was positioned toward the high-reliability side but was also dispersed into lower-readability regions, consistent with a “precise expert” archetype: informationally robust yet linguistically dense. In contrast, DeepSeek-v3.2 occupied a unique and practically significant niche. It attained near-top-tier reliability while consistently generating the most readable text (highest FRES: mean 48.23, SD 9.16; lowest FKGL: mean 9.95, SD 1.87) (Table 2), presenting a more balanced profile in the Figure 1 heatmap. This observation serves as a critical proof-of-concept: the reliability-readability trade-off is not an immutable law but rather a mitigable and measurable engineering challenge. The profound and consistent intermodel differences, confirmed by highly significant Friedman tests (Table 3; all *P*<.001) and detailed in the pairwise comparison heatmap (Figure 3), underscore that these performance characteristics are inherent to model architecture and training, not artifacts of query phrasing. The persistence of these performance gaps under our “zero-shot” prompting protocol is a key methodological insight. By forgoing system prompts or complex instructions to mimic a naive user’s interaction, as described in the LLMs and Query Process subsection of the Methods, we reveal the “out-of-the-box” capability landscape that patients actually face. The failure of all models to approach the readability target under these conditions indicates that intrinsic model propensity, not superficial user instruction, is the primary governor of output complexity. This finding challenges the notion that simple prompt engineering alone will suffice for clinical adoption and shifts the onus for improvement decisively onto model developers and tailored fine-tuning protocols. This reframes readability not as a downstream formatting issue but as a core design constraint that must be addressed at the model or fine-tuning level.

### Clinical Implications and Future Directions

These findings have practical implications for clinician-supervised use of LLMs in the education of patients with AMD. Patients’ understanding of AMD causes, pathophysiology, chronic progression, treatment options, and follow-up requirements can influence treatment expectations, adherence, timely care-seeking, and shared decision-making. Because LLM outputs may increasingly shape patients’ knowledge before or between clinical visits, their reliability and readability have practical implications for the education of patients with AMD. However, incomplete, overly complex, or unreliable explanations may mislead patients or delay appropriate care. Therefore, evaluating the reliability and readability of AI-generated AMD education is directly relevant to treatment-related decision-making outcomes. Direct, unsupervised provision of current LLM outputs to patients with AMD risks disseminating information that is inaccurate, incomprehensible, or both. Therefore, a pragmatic, 3-stage pathway for translation is warranted. In the context of AMD, such misalignment may concretely translate into misunderstandings regarding anti-VEGF treatment expectations, disease chronicity, or the distinction between neovascular and atrophic pathways.

The initial step involves evidence-based model selection for clinical settings exploring AI-drafted materials. Our data provide clear guidance: if the priority is reliability for subsequent expert editing, Grok 4 may be a suitable starting point. If the goal is a better-balanced starting point for communication, DeepSeek-v3.2 may be considered, although human simplification and clinical review would still be required [29].

Selection alone, however, is insufficient. Future research should move from passive benchmarking toward active intervention. Our study design provides the ideal framework for this: the curated 30-prompt AMD set (Textbox 1) and the comprehensive baseline performance data (Tables 1 and 2) can be used to rigorously test specific interventions. These include advanced prompt engineering strategies, such as chain-of-thought prompting for simplification, or fine-tuning protocols explicitly designed to close the quantified “readability gap,” for instance, by reducing the average FKGL of DeepSeek-v3.2 from 9.95 toward the target of 6, while vigilantly preserving reliability.

Ultimately, the most viable and safest near-term application is a synergistic human-AI workflow [30]. In this model, LLMs such as DeepSeek-v3.2 or Grok 4 act as powerful first-draft engines, significantly reducing clinician time spent on initial content creation [31]. This draft must then undergo mandatory review and literacy-sensitive simplification by a health care professional. This integrated workflow leverages AI’s scalability for efficiency while retaining irreplaceable human clinical judgment for safety, accuracy, and ultimate patient comprehension.

### Strengths and Limitations

#### Strengths

This study has several strengths. First, it provides a head-to-head comparison of 6 publicly accessible state-of-the-art conversational LLMs within a clinically specific ophthalmic context, rather than evaluating a single model or a general medical prompt set. Second, the 30-prompt benchmark was developed from both real-world patient search behavior and authoritative AMD clinical guidance, thereby improving the clinical relevance and patient-facing applicability of the evaluation. Third, the study jointly assessed informational reliability and linguistic readability using 4 established quality instruments and 6 readability formulas, allowing the reliability-readability gap to be quantified rather than described qualitatively. Fourth, all responses were independently evaluated by 2 senior ophthalmologists blinded to model identity, with adjudication of disagreements, strengthening the rigor and reproducibility of the scoring process. Finally, the zero-shot design reflects the initial, unoptimized interaction that a naïve patient may have with a publicly available LLM, providing a realistic baseline for future model selection, prompt optimization, and clinician-supervised deployment.

#### Limitations

This study has several limitations. First, this study evaluated 6 selected public web interface LLM responses and used a limited set of 30 AMD-related prompts. Therefore, the findings should not be generalized to all AMD-related patient information needs, retrieval-augmented answer engines, locally deployed open-source models, API-only systems, or medical-specialized models. This limitation was partly mitigated by selecting broadly accessible models from major international and Chinese developers and by deriving the prompt set from both real-world search behavior and authoritative AMD guidance.

Second, only 1 response was collected for each model-prompt pair under a zero-shot, single-turn design. Although this approach was intended to reflect the first answer a naïve patient might receive, it does not capture within-model stochastic variability, repeated regenerations, or multiturn patient-LLM interactions. Public web interfaces may also include hidden system prompts, interface-level settings, dynamic context handling, dynamic safety filters, and back-end updates that were not controllable by the investigators. Therefore, the findings should be interpreted as version- and interface-dependent snapshots rather than fixed estimates of model performance. Future studies should incorporate repeated regenerations, API-based controlled sampling where available, and multiturn dialogue designs to better quantify response variability, ecological validity, and reproducibility. An additional limitation concerns default interface-level retrieval behavior. Although dedicated retrieval-augmented answer engines were excluded, several raw outputs indicated that some evaluated public interfaces, particularly ChatGPT-5.1-auto and Qwen3-Max, displayed source links, tracking links, or bracketed web citations under default settings. This weakens any interpretation of the study as a comparison of strictly stand-alone model knowledge. Such interface-level retrieval or citation behavior may have influenced response breadth, apparent currency, attribution, and JAMA benchmark scores. Accordingly, our findings should be interpreted as default public interface performance snapshots rather than fixed estimates of base-model capability. Future studies should separately evaluate browsing-enabled and browsing-disabled modes, where available, and explicitly record whether web retrieval or citation display was enabled, reporting retrieval or citation display status for each model and, ideally, for each response. Although explicit model names, interface labels, and self-identifying boilerplate were removed before scoring, complete blinding cannot be guaranteed. Some reference URLs retained in the scoring materials may have contained model-identifying tracking parameters, such as UTM tags, which could have compromised complete blinding for responses from models whose interfaces generated such links. In addition, different LLMs may have recognizable stylistic patterns, response structures, or residual boilerplate language. Therefore, residual rater unblinding remains possible despite anonymization and standardized scoring procedures.

Third, all prompts and model outputs analyzed in this study were in English, and the readability formulas used here are primarily validated for English-language text. Although Chinese guideline–derived prompts were translated and reviewed before model querying, the findings may not directly apply to Chinese-language or other non-English patient education settings. In addition, readability scores were calculated using a single online calculator, which may introduce calculator-specific variation despite consistent application across all outputs.

Fourth, the prompt set was not composed exclusively of layperson-style patient education prompts. Several prompts were derived from clinical guidelines or included specialized AMD-related terminology, including prompts related to diagnostic coding or optical coherence tomography angiography–based management of nonexudative macular neovascularization. These clinician-adjacent prompts may have induced more technical responses and contributed to the high reading-grade levels observed. Readability estimates for responses containing alphanumeric diagnostic codes or list-based *ICD-10* (*International Classification of Diseases, Tenth Revision*) outputs may be mechanically distorted because such content can affect syllable counts, word counts, and sentence segmentation. Consequently, the findings should be interpreted as reflecting a mixed AMD information-seeking scenario rather than a pure layperson FAQ setting.

Fifth, hallucination was not prespecified as an independent analytic end point. In this study, hallucination was operationally understood as fabricated clinical facts, guideline-inconsistent recommendations, unsupported certainty regarding diagnosis or treatment, or nonverifiable references presented as evidence. Although the clinical raters evaluated reliability, transparency indicators, overall quality, and readability using DISCERN, EQIP, GQS, and JAMA criteria, they did not formally record hallucination frequency, perform claim-level fact checking, or assign binary hallucination labels to each response. Therefore, the present findings should not be interpreted as a direct estimate of hallucination risk, guideline concordance, or clinical safety. This limitation is clinically important because hallucinated AMD-related information could mislead patients about disease prognosis, urgency of anti-VEGF therapy, monitoring intervals, nutritional supplementation, or the distinction between neovascular and nonexudative disease. Future studies should incorporate prespecified hallucination detection, claim-level verification against current clinical guidelines, interrater agreement for hallucination labels, and model-level hallucination frequency reporting. In addition, the JAMA benchmark was originally developed for online health information rather than conversational AI. Because unprompted LLMs do not have conventional human authorship or financial disclosure structures, low JAMA scores should be interpreted as reflecting limited visible transparency and verifiability within model outputs rather than as direct evidence of poor factual accuracy or clinical unsafety.

### Conclusions

In this comparative evaluation of 6 publicly accessible LLMs, AMD-related patient-facing responses showed substantial model-dependent differences in informational reliability, transparency indicators, overall quality, and readability. No model met the recommended sixth-grade readability benchmark under zero-shot, single-turn prompting conditions, indicating that current LLM outputs remain insufficient as directly deployable education materials for patients with AMD. These findings support the need for clinician oversight, readability optimization, and further evaluation using repeated sampling, multiturn interactions, and claim-level accuracy assessment before LLM-generated AMD information is used in patient-facing settings.

## Supplementary material

10.2196/91016 Multimedia Appendix 1 Finalized age-related macular degeneration prompt set, query and collection protocol, and representative unedited raw outputs from 6 large language models.

## Abbreviations

AI: artificial intelligence
AMD: age-related macular degeneration
ARI: Automated Readability Index
CLI: Coleman-Liau Index
EQIP: Ensuring Quality Information for Patients
FKGL: Flesch-Kincaid Grade Level
FRES: Flesch Reading Ease Score
GFI: Gunning Fog Index
GQS: Global Quality Scale
*ICD-10*: *International Classification of Diseases, Tenth Revision*
JAMA: Journal of the American Medical Association benchmark criteria
LLM: large language model
SMOG: Simple Measure of Gobbledygook
UTM: Urchin Tracking Module
VEGF: vascular endothelial growth factor

## References

[1] Boopathiraj N, Wagner IV, Dorairaj SK, Miller DD, Stewart MW (2024). Recent updates on the diagnosis and management of age-related macular degeneration. Mayo Clin Proc Innov Qual Outcomes.

[2] Wong WL, Su X, Li X (2014). Global prevalence of age-related macular degeneration and disease burden projection for 2020 and 2040: a systematic review and meta-analysis. Lancet Glob Health.

[3] Wang H, Ie A, Chan T (2025). ChatGPT-4 for addressing patient-centred frequently asked questions in age-related macular degeneration clinical practice. Eye (Lond).

[4] Kufta AY, Djalilian AR (2025). Enhancing patient education with AI: a readability analysis of AI-generated versus American Academy of ophthalmology online patient education materials. J Clin Med.

[5] Fortuna J, Riddering A, Shuster L, Lopez-Jeng C (2020). Assessment of online patient education materials designed for people with age-related macular degeneration. BMC Ophthalmol.

[6] Will J, Gupta M, Zaretsky J, Dowlath A, Testa P, Feldman J (2025). Enhancing the readability of online patient education materials using large language models: cross-sectional study. J Med Internet Res.

[7] Eid K, Eid A, Wang D, Raiker RS, Chen S, Nguyen J (2024). Optimizing ophthalmology patient education via ChatBot-generated materials: readability analysis of AI-generated patient education materials and the American Society of Ophthalmic Plastic and Reconstructive Surgery patient brochures. Ophthalmic Plast Reconstr Surg.

[8] Aydin S, Karabacak M, Vlachos V, Margetis K (2024). Large language models in patient education: a scoping review of applications in medicine. Front Med (Lausanne).

[9] Huang G, Fang CH, Agarwal N, Bhagat N, Eloy JA, Langer PD (2015). Assessment of online patient education materials from major ophthalmologic associations. JAMA Ophthalmol.

[10] Prabhu AV, Gupta R, Kim C (2016). Patient education materials in dermatology: addressing the health literacy needs of patients. JAMA Dermatol.

[11] Patel PN, Patel PA, Ahmed H (2024). Assessment of the quality, accountability, and readability of online patient education materials for optic neuritis. Neuroophthalmology.

[12] Williams AM, Muir KW, Rosdahl JA (2016). Readability of patient education materials in ophthalmology: a single-institution study and systematic review. BMC Ophthalmol.

[13] Martin CA, Khan S, Lee R (2022). Readability and suitability of online patient education materials for glaucoma. Ophthalmol Glaucoma.

[14] Sandmann S, Hegselmann S, Fujarski M (2025). Benchmark evaluation of DeepSeek large language models in clinical decision-making. Nat Med.

[15] Builtjes L, Bosma J, Prokop M, van Ginneken B, Hering A (2025). Leveraging open-source large language models for clinical information extraction in resource-constrained settings. JAMIA Open.

[16] Sivarajkumar S, Kelley M, Samolyk-Mazzanti A, Visweswaran S, Wang Y (2024). An empirical evaluation of prompting strategies for large language models in zero-shot clinical natural language processing: algorithm development and validation study. JMIR Med Inform.

[17] Chinese Vitreo-Retina Society of Chinese Medical Association; Fundus Disease Group of Chinese Ophthalmologist Association (2023). Evidence-based guidelines for diagnosis and treatment of age-related macular degeneration in China (2023). Zhonghua Yan Ke Za Zhi.

[18] Vemulakonda GA, Bailey ST, Kim SJ (2025). Age-related macular degeneration preferred practice pattern®. Ophthalmology.

[19] Charnock D, Shepperd S, Needham G, Gann R (1999). DISCERN: an instrument for judging the quality of written consumer health information on treatment choices. J Epidemiol Community Health.

[20] Weil AG, Bojanowski MW, Jamart J, Gustin T, Lévêque M (2014). Evaluation of the quality of information on the Internet available to patients undergoing cervical spine surgery. World Neurosurg.

[21] Moult B, Franck LS, Brady H (2004). Ensuring quality information for patients: development and preliminary validation of a new instrument to improve the quality of written health care information. Health Expect.

[22] Hain T (2002). Improving the quality of health information: the contribution of C-H-i-Q. Health Expect.

[23] Silberg WM, Lundberg GD, Musacchio RA (1997). Assessing, controlling, and assuring the quality of medical information on the internet: caveant lector et viewor--Let the reader and viewer beware. JAMA.

[24] Smith EA, Senter RJ (1967). Automated Readability Index. AMRL TR.

[25] Yıldız HA, Söğütdelen E (2025). AI Chatbots as sources of STD information: a study on reliability and readability. J Med Syst.

[26] Eltorai AEM, Ghanian S, Adams CA, Born CT, Daniels AH (2014). Readability of patient education materials on the American Association for Surgery of Trauma website. Arch Trauma Res.

[27] van Ballegooie C, Hoang P (2021). Assessment of the readability of online patient education material from major geriatric associations. J Am Geriatr Soc.

[28] Nasra M, Jaffri R, Pavlin-Premrl D (2025). Can artificial intelligence improve patient educational material readability? A systematic review and narrative synthesis. Intern Med J.

[29] Wang W, Zhou Y, Fu J, Hu K (2025). Evaluating the performance of DeepSeek-R1 and DeepSeek-V3 versus OpenAI models in the Chinese National Medical Licensing Examination: cross-sectional comparative study. JMIR Med Educ.

[30] Ogut E (2025). Artificial intelligence in clinical medicine: challenges across diagnostic imaging, clinical decision support, surgery, pathology, and drug discovery. Clin Pract.

[31] Tripathi S, Sukumaran R, Cook TS (2024). Efficient healthcare with large language models: optimizing clinical workflow and enhancing patient care. J Am Med Inform Assoc.

