# Supplementary material for: Mapping the Reliability-Readability Gap in the Education of Patients With Age-Related Macular Degeneration Across 6 Large Language Models: Comparative Evaluation Study
Source: JMIR Med Inform. 2026 Jul 28;14:e91016. doi: 10.2196/91016 (PMC13411430; doi:10.2196/91016)
Supplement: Multimedia Appendix 1 [file medinform-v14-e91016-s001.docx]

**Multimedia Appendix 1**

**Finalized Prompt Set, Query Protocol, and Representative Raw Model Outputs**

This supplementary appendix provides the finalized prompts entered into the evaluated large language models (LLMs), the query and collection protocol, and representative unedited raw outputs used to support reproducibility of the study.

# **S1. Finalized Prompt Set**

The final AMD prompt set consisted of 30 English-language prompts derived from Google Trends, the 2023 Chinese AMD guideline, and the 2025 American Academy of Ophthalmology Preferred Practice Pattern. Google Trends-derived prompts were retained in their original search-query format rather than rewritten into complete questions because the study aimed to simulate real-world public-user information seeking under zero-shot conditions.

| **Prompt No.** | **Source** | **Prompt type** | **Final input entered into LLMs** |
| --- | --- | --- | --- |
| Q1 | Google Trends | Search-query prompt | macular degeneration icd 10 |
| Q2 | Google Trends | Search-query prompt | neovascular age-related macular degeneration |
| Q3 | Google Trends | Search-query prompt | neovascular |
| Q4 | Google Trends | Search-query prompt | exudative age-related macular degeneration |
| Q5 | Google Trends | Search-query prompt | nonexudative age-related macular degeneration |
| Q6 | Google Trends | Search-query prompt | age-related macular degeneration treatment |
| Q7 | Google Trends | Search-query prompt | macular degeneration treatment |
| Q8 | Google Trends | Search-query prompt | what is macular degeneration |
| Q9 | Google Trends | Search-query prompt | age related macular degeneration |
| Q10 | Google Trends | Search-query prompt | exudative macular degeneration icd 10 |
| Q11 | Google Trends | Search-query prompt | nonexudative age-related macular degeneration icd 10 |
| Q12 | Google Trends | Search-query prompt | choroidal neovascularization |
| Q13 | Google Trends | Search-query prompt | retinopathy |
| Q14 | Google Trends | Search-query prompt | age-related macular degeneration symptoms |
| Q15 | 2023 Chinese AMD guideline | Guideline-derived clinical prompt | What is the preferred diagnostic modality for neovascular AMD? |
| Q16 | 2023 Chinese AMD guideline | Guideline-derived clinical prompt | Should nutritional supplements be recommended for patients with early to intermediate AMD? |
| Q17 | 2023 Chinese AMD guideline | Guideline-derived clinical prompt | How should patients with AMD who exhibit a suboptimal initial response to anti-VEGF therapy be managed? |
| Q18 | 2023 Chinese AMD guideline | Guideline-derived clinical prompt | How should persistent pigment epithelial detachment after anti-VEGF therapy in AMD patients be managed? |
| Q19 | 2023 Chinese AMD guideline | Guideline-derived clinical prompt | How should “nonexudative” macular neovascularization (MNV) detected by OCTA in AMD patients be managed? |
| Q20 | 2023 Chinese AMD guideline | Guideline-derived clinical prompt | What is the appropriate follow-up protocol for patients with AMD? |
| Q21 | 2025 AAO PPP | Guideline-derived topic prompt | AMD Definition and Classification |
| Q22 | 2025 AAO PPP | Guideline-derived topic prompt | AMD Epidemiology and Risk Factors |
| Q23 | 2025 AAO PPP | Guideline-derived topic prompt | AMD Diagnosis and Monitoring |
| Q24 | 2025 AAO PPP | Guideline-derived topic prompt | Early and Intermediate AMD Management |
| Q25 | 2025 AAO PPP | Guideline-derived topic prompt | Neovascular AMD Anti-VEGF Therapy |
| Q26 | 2025 AAO PPP | Guideline-derived topic prompt | Geographic Atrophy in AMD |
| Q27 | 2025 AAO PPP | Guideline-derived topic prompt | AMD Treatment Protocols |
| Q28 | 2025 AAO PPP | Guideline-derived topic prompt | AMD Treatment Complications |
| Q29 | 2025 AAO PPP | Guideline-derived topic prompt | AMD Patient Management |
| Q30 | 2025 AAO PPP | Guideline-derived topic prompt | AMD Socioeconomic Considerations |

# **S2. Query and Collection Protocol**

All model outputs were collected between October 10 and November 25, 2025. The evaluated model identifiers or modes were recorded as displayed in the public interfaces during the study period: ChatGPT-5.1-auto, DeepSeek-v3.2, Gemini-2.5-Flash-Thinking, Grok 4, Claude-Sonnet 4.5, and Qwen3-Max.

All queries were conducted through the official publicly accessible web interfaces of the evaluated models. No separate retrieval-augmented answer engine, browser extension, plug-in, API call, automated script, or programmatic access method was used. However, default retrieval, browsing, or citation-display functions embedded in the evaluated public web interfaces were not manually disabled or standardized. Retrieval- or citation-related artifacts observed in raw outputs were retained for reproducibility. API-level generation parameters, including temperature, top-p, maximum token length, and frequency or presence penalties, were not displayed to or controllable by the investigators and were not manually set.

All Google Trends searches and LLM queries were conducted in private/incognito browsing mode after signing out of Google and other personal accounts. Browser cache and cookies were cleared before each session. This study did not involve YouTube video searches, video selection, or algorithmically recommended video content.

All prompts and outputs were in English. Each prompt was entered verbatim in a separate new session, without prior conversational history, additional context, custom instructions, user-specified system prompts, repeated prompt optimization, regeneration, or selective response replacement. One response was collected from each model for each prompt.

Before clinical scoring, model responses were transferred from the original model-specific files into a standardized scoring template and assigned anonymized response codes. Model names, file names, interface labels, logos, timestamps, ordering information, and any explicit self-identifying boilerplate text indicating the model name or developer were removed or redacted before rater review. This redaction was limited to model-identifying information and did not alter substantive clinical content, wording, recommendations, references, uncertainty statements, or safety-related statements. URL-level tracking parameters embedded within reference links, including UTM parameters, were not systematically scrubbed before scoring because references were otherwise preserved to avoid altering attribution-related content. Therefore, some reference URLs in the scoring materials may have retained model-identifying tracking strings.

# **S3. Representative Raw Model Outputs**

The following representative raw outputs were selected to illustrate the zero-shot responses generated by all six evaluated models. These examples include one search-query prompt, one common patient-facing treatment prompt, and one guideline-derived technical clinical prompt. Raw outputs are presented as plain text; only formatting was standardized for readability.

| **Representative prompt** | **Reason for inclusion** |
| --- | --- |
| Q1. macular degeneration icd 10 | Search-query style prompt derived from Google Trends; reflects real-world keyword-based public information seeking. |
| Q6. age-related macular degeneration treatment | Common patient-facing treatment-related prompt. |
| Q19. How should “nonexudative” macular neovascularization (MNV) detected by OCTA in AMD patients be managed? | Guideline-derived technical clinical prompt. |

## **S3.1 Representative Raw Outputs for Q1: “macular degeneration icd 10”**

### **ChatGPT-5.1-auto**

The ICD-10 codes for macular degeneration depend on the type and laterality of the condition. Here’s a breakdown:

Age-related macular degeneration (AMD)

H35.31 — Nonexudative (dry) age-related macular degeneration

H35.311 — Right eye

H35.312 — Left eye

H35.313 — Bilateral

H35.319 — Unspecified eye

H35.32 — Exudative (wet) age-related macular degeneration

H35.321 — Right eye

H35.322 — Left eye

H35.323 — Bilateral

H35.329 — Unspecified eye

Other macular degenerations

H35.30 — Unspecified macular degeneration

H35.34 — Toxic maculopathy (e.g., due to drugs)

H35.35 — Cystoid macular degeneration

H35.36 — Macular puckering

H35.37 — Macular hole

If you tell me whether you’re referring to dry vs. wet, or a specific type or cause, I can give you the most precise ICD-10 code for documentation or billing.

### **DeepSeek-v3.2**

Of course. Here is the information on ICD-10 codes for Macular Degeneration, broken down for clarity.

The core ICD-10 code category for macular degeneration is H35.3- (Degeneration of macula and posterior pole).

However, this is a "parent code" and requires an additional digit to specify the type and laterality (which eye is affected).

Most Common Codes for Macular Degeneration

Here are the specific codes you will use most often:

1. Age-Related Macular Degeneration (AMD)

This is the most common form and has its own subcategory.

H35.31- Age-related macular degeneration

H35.311 - Nonexudative age-related macular degeneration, right eye

H35.312 - Nonexudative age-related macular degeneration, left eye

H35.313 - Nonexudative age-related macular degeneration, bilateral

H35.321 - Exudative age-related macular degeneration, right eye

H35.322 - Exudative age-related macular degeneration, left eye

H35.323 - Exudative age-related macular degeneration, bilateral

Key Definitions:

Nonexudative (Dry AMD): The more common, slower-progressing form characterized by drusen and geographic atrophy.

Exudative (Wet AMD): The less common but more aggressive form, involving abnormal blood vessel growth and leakage.

2. Other Macular Degeneration (Non-Age-Related)

When the degeneration is not age-related, you use a different fourth character.

H35.30- Other macular degeneration (unspecified type)

H35.33- Cystoid macular degeneration

H35.34- Macular cyst, hole, or pseudohole

H35.36- Toxic maculopathy

These also require a 5th digit for laterality (e.g., H35.341 for cystoid macular degeneration, right eye).

Important Coding Guidelines

1. Laterality is Crucial: You must specify the right, left, or bilateral eye. Using an unspecified code (e.g., H35.30) is generally not accepted by payers if the eye is known.

2. Active Treatment: When a patient is receiving active treatment for Wet AMD (like anti-VEGF injections - Lucentis, Eylea, etc.), the Exudative AMD code (H35.32-) is the primary code used for the encounter.

3. Combination Codes: Some codes include the cause. For example, if the macular degeneration is a direct result of an injury, you would use a code from the injury chapter (S05.-) instead.

4. Documentation: The medical record must clearly document the type (Dry/Wet/Other) and the eye(s) affected to support the code selection.

Summary Table for Quick Reference

Condition; Type; ICD-10 Code

Age-Related (AMD); Dry (Nonexudative), Right Eye; H35.311

Age-Related (AMD); Dry (Nonexudative), Left Eye; H35.312

Age-Related (AMD); Dry (Nonexudative), Both Eyes; H35.313

Age-Related (AMD); Wet (Exudative), Right Eye; H35.321

Age-Related (AMD); Wet (Exudative), Left Eye; H35.322

Age-Related (AMD); Wet (Exudative), Both Eyes; H35.323

Other Macular Degeneration; Cystoid, Right Eye; H35.341

Disclaimer: This information is for educational purposes only. Medical coding is complex and requires careful review of the official ICD-10-CM guidelines and the patient's specific medical record. Always rely on a certified coder or the most current official coding resources for accurate billing and documentation.

### **Gemini-2.5-Flash-Thinking**

The ICD-10 codes for macular degeneration fall primarily under the category H35.3: Degeneration of macula and posterior pole.

Since ICD-10 coding often requires a high level of specificity for clinical and billing purposes (especially in the U.S. version, ICD-10-CM), you should use the most detailed code available.

Here are the main categories for age-related macular degeneration (AMD):

H35.30: Unspecified macular degeneration (Used when the type or laterality is not documented).

H35.31: Nonexudative age-related macular degeneration (Dry AMD) - This category has very specific codes based on the affected eye(s) and the stage of the disease (early, intermediate, or advanced atrophic).

Examples include:

H35.3111: Nonexudative age-related macular degeneration, right eye, early dry stage.

H35.3134: Nonexudative age-related macular degeneration, bilateral, advanced atrophic with subfoveal involvement.

H35.32: Exudative age-related macular degeneration (Wet AMD) - This category also has specific codes based on laterality and the status of choroidal neovascularization (CNV) (active, inactive, or inactive scar).

Examples include:

H35.3211: Exudative age-related macular degeneration, right eye, with active choroidal neovascularization.

H35.3232: Exudative age-related macular degeneration, bilateral, with inactive choroidal neovascularization.

To select the correct code, you must know:

1. Type: Is it Nonexudative (Dry) or Exudative (Wet) age-related macular degeneration?

2. Laterality: Which eye is affected (Right, Left, Bilateral, or Unspecified)?

3. Stage/Status: What is the stage of dry AMD (early, intermediate, advanced) or the CNV status for wet AMD (active, inactive, scar)?

### **Grok 4**

The ICD-10-CM code for unspecified macular degeneration is H35.30. This is the general code used when the type or specifics are not detailed.

Macular degeneration is often classified more specifically, particularly as age-related macular degeneration (AMD), which includes dry (nonexudative) and wet (exudative) forms. These require additional characters for laterality (right eye, left eye, bilateral, or unspecified) and stage (early, intermediate, advanced atrophic without/with subfoveal involvement, or unspecified). The base codes are as follows:

Type; Base ICD-10-CM Code; Notes Nonexudative age-related macular degeneration; H35.31xx; "xx" denotes laterality (1 = right, 2 = left, 3 = bilateral, 4 = unspecified) and stage (0 = unspecified, 1 = early, 2 = intermediate, 3 = advanced atrophic without subfoveal involvement, 4 = advanced atrophic with subfoveal involvement). Example: H35.3120 for left eye, stage unspecified. Exudative age-related macular degeneration; H35.32xx; Similar structure for "xx" as above, but includes active choroidal neovascularization (CNV) status (1 = with active CNV, 2 = with inactive CNV, 3 = with inactive scar). Example: H35.3230 for bilateral, stage unspecified. Cystoid macular degeneration; H35.35x; "x" for laterality (1 = right, 2 = left, 3 = bilateral, 9 = unspecified).

For precise coding in medical billing or documentation, consult the full ICD-10-CM guidelines or a healthcare professional, as codes can vary based on exact diagnosis details and annual updates.

### **Claude-Sonnet 4.5**

The ICD-10 codes for macular degeneration are:

Age-related macular degeneration (AMD):

H35.30 - Unspecified macular degeneration

H35.31 - Nonexudative age-related macular degeneration (dry AMD)

H35.311 - Nonexudative age-related macular degeneration, right eye

H35.312 - Nonexudative age-related macular degeneration, left eye

H35.313 - Nonexudative age-related macular degeneration, bilateral

H35.319 - Nonexudative age-related macular degeneration, unspecified eye

H35.32 - Exudative age-related macular degeneration (wet AMD)

H35.321 - Exudative age-related macular degeneration, right eye

H35.322 - Exudative age-related macular degeneration, left eye

H35.323 - Exudative age-related macular degeneration, bilateral

H35.329 - Exudative age-related macular degeneration, unspecified eye

Other macular degeneration codes:

H35.35 - Cystoid macular degeneration

H35.36 - Drusen (degenerative) of macula

H35.37 - Puckering of macula

H35.38 - Toxic maculopathy

The most commonly used codes are H35.31 (dry AMD) and H35.32 (wet AMD), with laterality specified when known.

### **Qwen3-Max**

The ICD-10 coding system includes several specific codes for different types of macular degeneration:

Unspecified macular degeneration is coded as H35.30 .

Nonexudative (dry) age-related macular degeneration, bilateral, is assigned code H35.313 .

Exudative (wet) age-related macular degeneration falls under H35.32, with laterality specified (e.g., H35.322 for the left eye) [[5], [9]].

There are also codes for other forms, such as cystoid macular degeneration (e.g., H35.353 for bilateral) and drusen of the macula (e.g., H35.361 for the right eye) .

These codes are part of the ICD-10-CM chapter on "Diseases of the eye and adnexa," specifically under "Other retinal disorders" [[4], [7]]. Accurate coding often requires specifying both the type (dry vs. wet) and the affected eye(s) .

## **S3.2 Representative Raw Outputs for Q6: “age-related macular degeneration treatment”**

### **ChatGPT-5.1-auto**

Treatment for age-related macular degeneration (AMD) depends on whether it is the “dry” (atrophic / non-neovascular) form or the “wet” (neovascular) form. I’ll summarize the current standard therapies, emerging options, limitations, and what is under research. (Note: always consult a retinal specialist / ophthalmologist for personalized advice.)

Background: Dry vs Wet AMD

Dry AMD is more common; it is characterized by accumulation of drusen (yellow deposits) under the retina, thinning of retinal pigment epithelium (RPE), and in advanced cases — geographic atrophy (GA), a loss of photoreceptors and supporting layers.

Wet AMD involves abnormal, leaky blood vessel growth (choroidal neovascularization, CNV) under the retina, which causes bleeding, fluid, and scarring, often more rapidly damaging central vision.

Because their mechanisms differ, treatments differ too.

Established Treatments

For Wet AMD

These are the backbone of current therapy:

1. Anti-VEGF (vascular endothelial growth factor) intravitreal injections

— Drugs like ranibizumab (Lucentis), aflibercept (Eylea), bevacizumab (Avastin, off-label), brolucizumab (Beovu), faricimab (Vabysmo) are used to block VEGF, reducing abnormal vessel growth and leakage. ([nei.nih.gov][1])

— You typically receive injections monthly or at varying intervals depending on response. ([nei.nih.gov][1])

— A newer “high dose” formulation of Eylea is being explored to extend the interval between injections. ([aao.org][2])

— Dual-action or longer-acting agents (e.g. faricimab) are designed to reduce treatment frequency. ([macularsociety.org][3])

2. Photodynamic therapy (PDT)

— A photosensitizing agent (verteporfin) is injected, then a low-power laser “activates” it locally to obliterate abnormal vessels. Sometimes used in combination with anti-VEGF therapy. ([Cleveland Clinic][4])

3. Laser photocoagulation

— This older method uses thermal laser to seal leaking vessels. Its use has declined because of collateral damage risk. ([Cleveland Clinic][4])

Although these therapies don’t cure AMD, they can often halt or slow further vision loss, and in some patients, achieve modest vision improvement. ([PMC][5])

Risks (which your ophthalmologist will monitor for) include infection (endophthalmitis), retinal detachment, increased intraocular pressure, cataract formation, etc. ([Cleveland Clinic][4])

For Dry AMD (non-neovascular)

Historically, this has been more challenging to treat. Until recently, there was no effective therapy to reliably slow or reverse dry AMD or geographic atrophy in most patients. ([nhs.uk][6])

However, recent advances are changing that:

Complement pathway inhibitors

Two drugs approved in 2023 by the U.S. FDA for geographic atrophy (a late stage of dry AMD):

Pegcetacoplan (brand name Syfovre) — a complement C3 inhibitor injected intravitreally. ([aao.org][2])

Avacincaptad pegol (brand name Izervay) — a complement C5 inhibitor for GA. ([Verywell Health][7])

These slow the progression of geographic atrophy (i.e., reduce the rate at which retinal cells die) but do not restore cells already lost. ([PMC][8])

Photobiomodulation (light therapy)

A noninvasive approach using specific wavelengths of light (often near-infrared) to stimulate cellular repair or reduce oxidative stress. The FDA has recently authorized a form of light therapy for dry AMD. ([aao.org][9])

Cell / stem cell therapy and RPE transplantation

Investigational: the idea is to replace damaged retinal pigment epithelium (RPE) or photoreceptor support cells. For example, OpRegen (RG6501) is an allogeneic RPE cell therapy administered via subretinal injection in trials for GA. ([维基百科][10])

Gene therapy

Attempts are underway to deliver genes that produce protective or anti-complement proteins within the retina, reducing the need for repeated injections. For example, experimental gene therapies are being tested especially in wet AMD. ([health.ucdavis.edu][11])

Other agents in research

Sefaxersen — an antisense oligonucleotide targeting complement factor B (CFB) — under study for geographic atrophy. ([维基百科][12])

Combined or multi-target strategies are being explored to address oxidative stress, inflammation, metabolic stress, etc. ([PMC][8])

Supportive measures / low vision aids

Because damage in dry AMD accumulates slowly, there is emphasis on nutritional / lifestyle support (see below), and use of magnifiers, special glasses, contrast-enhancing devices, electronic aids, etc. ([nhs.uk][6])

Adjunctive & Lifestyle Strategies

While these are not treatments per se, they can help slow progression or reduce risk factors:

Nutritional supplements (AREDS / AREDS2 formulation):

A combination of vitamins and minerals (e.g., vitamins C, E, zinc, copper, lutein, zeaxanthin) has been shown (in the Age-Related Eye Disease Study) to reduce the risk of progression from intermediate to advanced AMD in some patients.

(Note: not effective for everyone and not a cure.)

This is more for dry AMD or early AMD. ([PMC][8])

Smoking cessation

Smoking is a strong risk factor; quitting is one of the best modifiable actions. ([PMC][8])

Diet and antioxidants

Diets rich in leafy greens, fish (omega-3s), and antioxidants may support retinal health. ([PMC][8])

Control of cardiovascular risk factors

Hypertension, hyperlipidemia, obesity, and poor vascular health may exacerbate AMD risk. ([PMC][8])

Regular monitoring

Frequent eye exams, optical coherence tomography (OCT), and monitoring of early changes help ophthalmologists detect progression (especially onset of wet AMD) early when interventions are more effective.

Emerging / Future Directions & Limitations

Many of the newer therapies (complement inhibitors, gene therapy, cell transplantation) do not yet restore lost vision; they aim to slow or halt further degeneration. ([PMC][8])

There remain challenges in delivering therapies safely, achieving long-term expression, avoiding inflammation or immune reactions, and dealing with heterogeneity among patients. ([PMC][8])

Some therapies in trials may take years to reach approval in many countries. ([BrightFocus Foundation][13])

A gene therapy approach for wet AMD is under study, attempting to allow the eye to produce its own anti-VEGF agents, reducing the need for repeated injections. ([health.ucdavis.edu][11])

Implants like the Port Delivery System (PDS, Susvimo) that continuously release anti-VEGF over months are being refined to reduce injection burden. ([macularsociety.org][3])

If you like, I can pull up which treatments are available or approved in Singapore / Southeast Asia, what the cost is, and what might be accessible to you. Would you like me to do that for your region?

[1]: https://www.nei.nih.gov/learn-about-eye-health/eye-conditions-and-diseases/age-related-macular-degeneration/treatments-wet-amd-advanced-neovascular-amd?utm_source=chatgpt.com "Treatments for Wet AMD (Advanced Neovascular AMD)"

[2]: https://www.aao.org/eye-health/tips-prevention/promising-new-treatments-amd?utm_source=chatgpt.com "New Treatments for Age-Related Macular Degeneration"

[3]: https://www.macularsociety.org/diagnosis-treatment/treatments/new-treatments/?utm_source=chatgpt.com "New treatments in the pipeline - Macular Society"

[4]: https://my.clevelandclinic.org/health/diseases/15246-macular-degeneration?utm_source=chatgpt.com "Macular Degeneration: Symptoms, Diagnosis & Treatment"

[5]: https://pmc.ncbi.nlm.nih.gov/articles/PMC3973093/?utm_source=chatgpt.com "Recent developments in the treatment of age-related macular ..."

[6]: https://www.nhs.uk/conditions/age-related-macular-degeneration-amd/treatment/?utm_source=chatgpt.com "Age-related macular degeneration (AMD) - Treatments - NHS"

[7]: https://www.verywellhealth.com/macular-degeneration-emerging-treatments-5071096?utm_source=chatgpt.com "Macular Degeneration Breakthroughs: Emerging Treatments"

[8]: https://pmc.ncbi.nlm.nih.gov/articles/PMC9414333/?utm_source=chatgpt.com "Recent Advances in Age-Related Macular Degeneration Therapies"

[9]: https://www.aao.org/eye-health/news/light-therapy-photobiomodulation-dry-amd-ga?utm_source=chatgpt.com "FDA Authorizes Light Therapy for Dry AMD"

[10]: https://en.wikipedia.org/wiki/OpRegen?utm_source=chatgpt.com "OpRegen"

[11]: https://health.ucdavis.edu/news/headlines/experimental-gene-therapy-for-wet-age-related-macular-degeneration-offers-promise-for-older-adults/2024/01?utm_source=chatgpt.com "Experimental gene therapy for wet age-related ... - UC Davis Health"

[12]: https://en.wikipedia.org/wiki/Sefaxersen?utm_source=chatgpt.com "Sefaxersen"

[13]: https://www.brightfocus.org/resource/emerging-treatments-offer-new-hope-for-dry-and-wet-age-related-macular-degeneration/?utm_source=chatgpt.com "Emerging Treatments Offer New Hope for Dry and Wet Age-Related ..."

### **DeepSeek-v3.2**

The treatment for age-related macular degeneration (AMD) depends on whether you have the "dry" (atrophic) or "wet" (neovascular) form of the disease. Wet AMD has well-established, effective treatments, while research for dry AMD is rapidly advancing, with a key focus on slowing progression .

The table below summarizes the current and emerging treatment landscape for AMD.

Form of AMD; Established Treatments; Emerging Treatments & Research Focus

Wet (Neovascular) AMD; Anti-VEGF Injections (standard care) :<br>• Ranibizumab (Lucentis)<br>• Aflibercept (Eylea)<br>• Biosimilars (e.g., Aflibercept-boav/EYDENZELT) <br>• Conbercept (Lumitin) <br><br>Adjunctive/Laser Therapies :<br>• Photodynamic Therapy (PDT)<br>• Laser Photocoagulation; Next-Generation Therapies :<br>• Drugs targeting pathways beyond VEGF-A (e.g., VEGF-C, VEGF-D, Ang-2)<br>• Sozinibercept, a VEGF-C/D inhibitor, shows promise in clinical trials when combined with standard anti-VEGF therapy .

Dry (Atrophic) AMD; Management & Supplements :<br>• High-dose antioxidant vitamins and zinc (AREDS2 formula) to slow progression in intermediate stages .<br>• Low vision aids .; Investigational Drugs :<br>• Complement inhibitors targeting the immune system.<br>• Other agents addressing oxidative stress, inflammation, and neuroprotection.<br>Note: There are currently no proven therapies to reverse geographic atrophy, but this is an active area of clinical research .

💡 Key Treatment Considerations

Treatment Goals Vary: For wet AMD, the goal of anti-VEGF therapy is to inhibit new blood vessel growth, reduce leakage, and stabilize or improve vision . For dry AMD, the current goal is to slow disease progression through supplements and manage vision loss with adaptive tools .

Wet AMD Requires Ongoing Treatment: Anti-VEGF therapy is not a cure. It involves regular injections into the eye, and despite its high effectiveness, some patients may experience variable responses or require frequent treatments .

Consult a Specialist: Diagnosis and management of AMD require a retina specialist. They can perform the necessary exams (like OCT and angiography) and create a personalized treatment plan .

I hope this overview helps you understand the current options for age-related macular degeneration. If you are comfortable sharing, knowing whether you or someone you know has been diagnosed with the wet or dry form can help me provide more specific information.

### **Gemini-2.5-Flash-Thinking**

Age-related Macular Degeneration (AMD) treatment depends on the stage and type of the disease (dry or wet AMD). While there is currently no cure, treatments and lifestyle changes can help slow progression and manage vision loss.

Here is a breakdown of the treatments for the different types of AMD:

1. Dry Age-Related Macular Degeneration (AMD)

Dry AMD is the most common form, and for the earlier stages, the focus is on slowing progression.

Nutritional Supplements (AREDS/AREDS2 Formula): For people with intermediate or advanced dry AMD, a specific high-dose combination of antioxidant vitamins and minerals has been shown to slow the progression to advanced AMD. The formula typically includes Vitamin C, Vitamin E, Lutein, Zeaxanthin, Zinc, and Copper. Your eye specialist will advise if these are right for you.

Targeted Treatments for Geographic Atrophy (Advanced Dry AMD): Newer FDA-approved treatments, such as certain complement inhibitors (e.g., pegcetacoplan and avacincaptad pegol), are available as eye injections to slow the progression of geographic atrophy (GA), a late stage of dry AMD.

Low Vision Aids and Rehabilitation: For advanced vision loss, low vision specialists can recommend devices (like magnifiers or electronic aids) and techniques to help patients make the most of their remaining vision.

Lifestyle Changes: Quitting smoking, eating a healthy diet rich in leafy green vegetables and fish, maintaining a healthy weight, and exercising regularly are recommended to reduce the risk of progression.

2. Wet Age-Related Macular Degeneration (AMD)

Wet AMD is less common but usually causes faster, more severe vision loss. The primary treatment is to stop the growth of abnormal, leaky blood vessels.

Anti-VEGF Injections (Angiogenesis Inhibitors): This is the most common and effective treatment. Medications are injected directly into the eye (intravitreal injections) to block Vascular Endothelial Growth Factor (VEGF), a protein that causes new, abnormal blood vessels to grow. Examples of these drugs include:

Aflibercept (Eylea, Eylea HD)

Ranibizumab (Lucentis)

Bevacizumab (Avastin - often used off-label)

Brolucizumab (Beovu)

Faricimab (Vabysmo)

These injections are typically given at regular intervals, which may vary depending on the medication and individual response (e.g., every 4 to 16 weeks).

Photodynamic Therapy (PDT): A two-step treatment where a light-sensitive drug is injected into a vein and then activated by a "cold" laser aimed at the retina to damage the abnormal blood vessels. It is used less often now but may be combined with anti-VEGF injections in specific cases.

Laser Photocoagulation: An older treatment that uses a high-energy laser to destroy abnormal blood vessels. It is rarely used today because it can also damage healthy tissue, resulting in a blind spot.

Emerging and Experimental Treatments

Research is ongoing for new treatments, especially for dry AMD:

Sustained-Release Drug Delivery Systems: Devices implanted in the eye (like the port delivery system) or long-acting injections are being developed to reduce the frequency of anti-VEGF injections for wet AMD.

Gene Therapy: Experimental treatments aim to introduce a gene into the eye so the eye's cells can continuously produce their own anti-VEGF medicine, potentially requiring only a single injection.

Stem Cell Therapy: Research is exploring using stem cells to replace damaged retinal cells in both dry and wet AMD.

It is important to have regular eye exams to detect AMD early. If you notice any sudden changes in your vision, see an eye specialist (optometrist or ophthalmologist) immediately.

### **Grok 4**

Overview of Age-Related Macular Degeneration (AMD)

Age-related macular degeneration is a common eye condition that affects the macula, the part of the retina responsible for central vision. It primarily occurs in people over 50 and can lead to blurred or distorted vision. There are two main types: dry AMD (the more common, early-stage form) and wet AMD (a more advanced form involving abnormal blood vessel growth). While there's no cure for AMD, treatments can slow progression, manage symptoms, and in some cases, improve vision. Always consult an eye care professional for personalized advice, as treatment depends on the type, stage, and individual health factors.

Treatments for Dry AMD

Dry AMD progresses slowly and involves the buildup of drusen (yellow deposits) under the retina. The focus is on slowing progression rather than reversing damage.

Nutritional Supplements: A specific formula from the Age-Related Eye Disease Study (AREDS or AREDS2) includes vitamins C and E, beta-carotene (or lutein/zeaxanthin in AREDS2 for non-smokers), zinc, and copper. These can reduce the risk of progression to advanced AMD by about 25% in people with intermediate or advanced dry AMD in one eye.

Lifestyle Changes: Quitting smoking, maintaining a healthy diet rich in leafy greens, fish, and antioxidants, controlling blood pressure and cholesterol, and protecting eyes from UV light with sunglasses. Regular exercise and weight management may also help.

Monitoring and Low-Vision Aids: Regular eye exams to track progression. For vision loss, devices like magnifiers, special glasses, or apps can assist daily activities. No injections or surgeries are typically used for dry AMD alone.

Treatments for Wet AMD

Wet AMD is less common but more aggressive, caused by leaky blood vessels under the retina. Treatments aim to stop or reduce vessel growth and leakage.

Anti-VEGF Injections: The primary treatment involves injecting drugs into the eye to block vascular endothelial growth factor (VEGF), which promotes abnormal vessel growth. Common medications include:

Aflibercept (Eylea)

Ranibizumab (Lucentis)

Faricimab (Vabysmo, a newer bispecific antibody that targets both VEGF and angiopoietin-2 for potentially longer-lasting effects)

Bevacizumab (Avastin, an off-label option)

These are given every 4-12 weeks initially, then as needed. They can stabilize or improve vision in about 90% of cases.

Photodynamic Therapy (PDT): Involves injecting a light-sensitive drug (verteporfin) into the arm, followed by laser activation in the eye to seal leaking vessels. It's less common now but used for certain cases, often combined with anti-VEGF.

Laser Photocoagulation: A laser seals abnormal blood vessels. It's rarely used today due to potential retinal damage but may be an option for vessels away from the macula's center.

Emerging Treatments: Research includes gene therapy, stem cell implants, and longer-acting anti-VEGF formulations to reduce injection frequency. For example, some patients may qualify for clinical trials of sustained-release implants.

Treatment Type; Best For; Frequency/Method; Potential Side Effects Anti-VEGF Injections; Wet AMD; Every 4-12 weeks, injected into the eye; Eye irritation, floaters, rare infection or bleeding Nutritional Supplements (AREDS2); Dry AMD (intermediate/advanced); Daily oral pills; Upset stomach, copper/zinc interactions Photodynamic Therapy; Wet AMD (specific cases); One-time or repeated sessions with laser; Temporary light sensitivity, skin reactions Laser Photocoagulation; Wet AMD (limited use); Outpatient procedure; Vision loss in treated area, scarring

Early detection through regular eye exams is crucial, especially if you have risk factors like family history, smoking, or obesity. If you're experiencing symptoms like central vision blurriness or straight lines appearing wavy, see an ophthalmologist promptly.

### **Claude-Sonnet 4.5**

Age-related macular degeneration (AMD) is a leading cause of vision loss in older adults. Treatment depends on the type:

Dry AMD (Geographic Atrophy)

Complement inhibitors: Pegcetacoplan (Syfovre) and avacincaptad pegol (Izervay) - the first FDA-approved treatments for geographic atrophy, given as intravitreal injections to slow progression

Nutritional supplements: AREDS2 formula (vitamins C, E, zinc, copper, lutein, and zeaxanthin) can slow progression in intermediate to advanced stages

Lifestyle modifications: Stop smoking, maintain healthy diet, protect eyes from UV light

Wet AMD (Neovascular)

Anti-VEGF injections (most common treatment):

Aflibercept (Eylea, Eylea HD)

Ranibizumab (Lucentis)

Brolucizumab (Beovu)

Bevacizumab (Avastin, off-label)

Faricimab (Vabysmo) - targets both VEGF and Ang-2

Photodynamic therapy (PDT): Verteporfin with laser, now rarely used as first-line treatment

Laser photocoagulation: Rarely used today, mainly for specific lesion types

Emerging Treatments

Gene therapy trials

Stem cell research

Port delivery systems for sustained drug release

Early detection is crucial - regular eye exams with dilated fundus examination and OCT imaging help catch AMD early when treatment is most effective. If you notice distorted vision, dark spots, or difficulty with central vision, see an ophthalmologist promptly.

Would you like more details about any specific treatment option?

### **Qwen3-Max**

Age-related macular degeneration (AMD) is a leading cause of vision loss in people over 50 and is broadly categorized into two types: dry (atrophic) and wet (neovascular) AMD . While there is currently no cure for AMD, several treatment options are available to slow progression and preserve vision, depending on the type and stage of the disease .

Treatments for Wet AMD:

Anti-VEGF injections are the standard of care. These medications—including aflibercept (Eylea), ranibizumab (Lucentis), faricimab (Vabysmo), and bevacizumab (Avastin)—are injected directly into the vitreous of the eye to block vascular endothelial growth factor (VEGF), which drives abnormal blood vessel growth in wet AMD [[3], [4], [6]].

These treatments can stabilize or even improve vision in many patients, though they are not a cure and require repeated injections [[3], [6]].

Photodynamic therapy and laser photocoagulation are older or adjunctive approaches that may be used in specific cases to destroy abnormal blood vessels [[1], [2]].

Treatments for Dry AMD:

There is no approved treatment to reverse dry AMD, but nutritional supplements (such as the AREDS2 formulation containing vitamins C and E, zinc, copper, lutein, and zeaxanthin) may help slow progression in intermediate to advanced cases [[2], [5]].

Vision aids and lifestyle modifications (e.g., smoking cessation, UV protection, and a healthy diet) are also recommended [[5], [10]].

Overall, early detection and regular monitoring are crucial, as timely intervention—especially for wet AMD—can significantly impact visual outcomes .

## **S3.3 Representative Raw Outputs for Q19: “How should “nonexudative” macular neovascularization (MNV) detected by OCTA in AMD patients be managed?”**

### **ChatGPT-5.1-auto**

The discovery of nonexudative (“subclinical” or “quiescent”) macular neovascularization (MNV) by OCT-angiography (OCTA) in an AMD patient raises important questions, but the current consensus is cautious: there is no established evidence-based therapy to treat such lesions before exudation. Rather, careful surveillance with multimodal imaging is the prevailing approach. Below is a summary of what is known and how to manage — including caveats, risk stratification, and when to consider intervention.

What is nonexudative MNV?

Nonexudative MNV refers to neovascular complexes (usually under the RPE, i.e. type 1) that are visible on OCTA (or ICGA) but do not exhibit clinical signs of leakage or fluid on structural OCT or FA/ICGA.

These lesions are often asymptomatic and may exist for months to years without causing vision loss.

Their prevalence in fellow eyes of patients with exudative AMD ranges from ~6 % to 27 % in various studies.

Importantly, nonexudative MNV is a risk factor (a “preclinical” stage) for future conversion to exudative disease.

Interestingly, there is some (still preliminary) evidence that nonexudative MNV may slow the growth of adjacent geographic atrophy (GA), possibly by supplying some perfusion to the outer retina.

Natural history & risk of conversion

Because management decisions depend heavily on the chance that the nonexudative lesion will convert to exudation, it's worth summarizing what is known:

Reported conversion (to exudative AMD) rates are quite variable — from ~20 % up to ~80 % in follow-up periods of 6 months to 2 years, depending on population, imaging method, lesion size, and follow-up intensity.

In a recent larger cohort (241 fellow eyes), 16.6 % had nonexudative MNV at baseline, and 32.5 % of those progressed to exudation over ~12.6 months (range 2.6–22.4 months).

Nature

A key risk factor appears to be growth of the MNV complex (for example, ≥ 50 % area increase within a year). Eyes with growing MNV had a significantly higher and earlier rate of exudation vs stable lesions.

Also, presence of a shallow irregular RPE elevation (SIRE) or double-layer sign (DLS) at baseline has been associated with a higher risk of conversion to exudation.

Other possible imaging biomarkers under investigation include lesion vascular density, the presence of a hypointense “halo” around the lesion, changes in choriocapillaris perfusion, or structural changes over time.

In one study, eyes classified as having a “growing” nonexudative MNV had a mean time to exudation of ~13.6 months, versus ~31.1 months in “stable” lesions.

Thus, we can stratify nonexudative MNV lesions roughly into “stable” vs “active/growing” phenotypes, which helps in planning follow-up.

Management principles

Given the current evidence, here is how most retinal specialists manage nonexudative MNV in AMD:

Do not treat preemptively with anti-VEGF in the absence of exudation.

There is no randomized trial evidence that prophylactic anti-VEGF therapy delays or prevents conversion to exudative disease in nonexudative MNV, and the risks of overtreatment (cost, injection risk, possible impact on choroid) argue against this approach.

Reviews and expert opinion consistently recommend careful observation until signs of exudation appear (fluid, hemorrhage, leakage).

Retina Today

One review states: “subclinical non-exudative lesions should not be treated until symptomatic exudation develops.”

Close surveillance with regular multimodal imaging

Because the risk of conversion is real, these eyes merit more frequent monitoring than “ordinary” dry AMD eyes. Key components:

OCTA (ideally swept-source OCTA) to monitor changes in lesion size, vascular morphology, density, and new flow patterns.

Structural OCT (B-scans) to detect new subretinal or intraretinal fluid or pigment epithelial detachments (PEDs).

OCT B-scans with flow overlay / cross-sectional OCTA overlay can help detect subtle exudation onset.

Optional FA / ICGA in ambiguous cases or when OCTA is inconclusive, especially if new symptoms arise.

Assessment interval: Many would follow every 2–3 months (or more frequently if signs of growth), but frequency may be tailored to lesion behavior (e.g. growing lesions demand more frequent follow-up).

Risk stratification & personalized monitoring

Use imaging biomarkers to adjust the intensity of surveillance:

If lesion is stable (no enlargement, stable appearance) → standard surveillance (e.g. every 3 months).

If lesion is growing, or new features (e.g. increased vascular density, halo changes, signs of increased activity) → closer follow-up (e.g. every 1–2 months).

If exudation is detected (fluid, hemorrhage), then manage as exudative AMD with anti-VEGF.

Some authors propose flagging lesions that cross a threshold size or grow rapidly as “pre-active,” though there is no consensus threshold.

Educate patient on symptoms

Because conversion may occur between scheduled visits, instruct patients to report new symptoms such as distortion (metamorphopsia), new blur, or scotoma immediately, which should trigger earlier re-imaging.

Be cautious about overtreatment and unwarranted interventions

In absence of exudation, aggressive interventions may carry risk (e.g. atrophy from repeated anti-VEGF, injection-related complications). Also, the possible protective effect of nonexudative MNV (delaying GA) is a consideration.

Research context / clinical trials

Because the field is evolving, enrollment in observational cohorts or trials (where available) should be considered. In the future, trials may explore prophylactic or low-dose therapy in certain high-risk lesions.

Summary: How you might manage it in practice

Putting the above into a clinical algorithm:

Scenario Action / Approach

Nonexudative MNV identified (no fluid, no symptoms) Do not treat immediately; begin enhanced surveillance

Lesion is stable (no growth, morphology unchanged) Monitor every ~3 months with OCT + OCTA, structural scans

Lesion shows growth, increasing vascular signals, or suspicious features Increase surveillance frequency (e.g. every 1–2 months)

Symptoms or structural OCT signs of exudation appear (fluid, hemorrhage) Initiate anti-VEGF therapy per standard exudative AMD protocol

Uncertain findings, ambiguous imaging Consider FA / ICGA and expert review

In selecting follow-up intervals and thresholds for action, individual patient factors matter: fellow eye history, visual risk, patient reliability for visits, imaging quality, and comorbidities.

Caveats, unknowns, and future directions

There is no prospective randomized trial proving benefit (or harm) of treating nonexudative MNV.

Many imaging biomarkers (growth rate, vascular density, halo signs) are promising but not yet fully validated for decision-making.

The “protective” effect (slowing GA) of nonexudative MNV remains speculative, and may not apply to all lesions or over the long term.

It is possible that in the future, particular high-risk lesions may be candidates for early intervention (e.g. low-dose or pro re nata anti-VEGF), but that remains investigational.

The imaging quality and segmentation errors in OCTA (especially under drusen, pigment changes, or atrophy) demand expert review and careful interpretation.

### **DeepSeek-v3.2**

The management of "nonexudative" macular neovascularization (MNV) detected by Optical Coherence Tomography Angiography (OCTA) in AMD patients is an evolving area in ophthalmology. Current expert consensus and clinical guidelines generally recommend observation over immediate treatment, but with close and active monitoring for the onset of exudation.

The table below summarizes the core principles for managing nonexudative MNV based on current guidelines and research.

Management Aspect; Recommended Action

💡 Overall Strategy; Active Observation (No immediate anti-VEGF injection)

📋 Diagnostic Confirmation; Use OCTA to confirm MNV type and structure; quantitative analysis of vessel features may be used.

👁️ Monitoring & Follow-up; Regular monitoring with both structural OCT and OCTA for early signs of exudation (e.g., fluid).

💧 Treatment Trigger; Initiate anti-VEGF therapy upon conversion to exudative MNV.

🔍 Key Concepts and Monitoring Strategies

Understanding the following concepts is crucial for effective management:

Understanding "Nonexudative MNV": This term refers to the presence of abnormal new blood vessels beneath the retina that have been detected by the high sensitivity of OCTA, but which are not currently leaking fluid or causing swelling. This condition was previously often described as "treatment-naive" or "quiescent" MNV. The latest Japanese clinical guidelines for nAMD have integrated OCTA into the diagnostic process and moved away from older terminology like "wet/dry" AMD.

The Role of Quantitative OCTA: Advanced OCTA analysis can provide more than just an image. It can quantify specific biomarkers of the MNV complex, such as its vessel density, surface area, and structural complexity (fractal dimension). Research indicates that these quantitative features may help predict the risk of an MNV becoming exudative, allowing for more personalized monitoring intervals.

Implementing a Monitoring Schedule: While a universal schedule is not standardized, the principle is consistent. Patients should be monitored periodically with both structural OCT (to check for fluid) and OCTA (to monitor for changes in the MNV itself). Any decision to extend the time between follow-up visits should be based on a stable appearance over multiple examinations.

💡 Future Directions

Research is actively exploring how to best manage this condition:

AI-Powered Analysis: Deep learning models are being developed to automatically and accurately segment and quantify MNV on OCTA images. This technology could reduce human error and provide highly objective data for risk stratification.

Advanced Therapeutics: For cases that do become exudative and prove difficult to treat, newer agents like faricimab, which inhibits both VEGF and Ang-2 pathways, have shown efficacy in extending treatment intervals for resistant cases.

I hope this overview is helpful for your clinical understanding. Should you require clarification on any of the specific diagnostic criteria or treatment protocols mentioned, please feel free to ask.

### **Gemini-2.5-Flash-Thinking**

The general consensus for managing "nonexudative" macular neovascularization (MNV) detected by OCTA in patients with Age-Related Macular Degeneration (AMD) is close observation and monitoring, without immediate anti-VEGF treatment.

Here is a breakdown of the management strategy and rationale:

1. Close Monitoring and Observation

No immediate anti-VEGF treatment: Nonexudative MNV (also called subclinical or quiescent MNV) is currently not recommended for treatment with anti-VEGF injections. Randomized controlled trials have not shown a benefit in preventing exudation or vision loss with prophylactic anti-VEGF therapy in these high-risk eyes.

High Risk of Conversion: Although asymptomatic and nonexudative, these lesions are a strong predictor and precursor for conversion to exudative (or "wet") AMD, which does require anti-VEGF treatment. Conversion rates vary but can be significant (e.g., 20% to 80% over 6 months to 2 years).

Monitoring Schedule: Patients should be followed closely in the clinic, typically every 1 to 3 months (though specific intervals may vary by clinician/protocol).

Home Monitoring: Patients should be educated on self-monitoring with an Amsler grid and to report any new symptoms immediately, such as:

Decreased or blurred vision

Metamorphopsia (distortion of straight lines)

The appearance of a scotoma (blind spot)

2. Treatment Indication: Conversion to Exudation

The decision to initiate anti-VEGF therapy is generally triggered by the conversion to exudative MNV.

Signs of Exudation (Fluid) on Structural OCT:

Intraretinal fluid (IRF)

Subretinal fluid (SRF)

Fluid beneath the retinal pigment epithelium (RPE)

3. Imaging Biomarkers for Increased Risk

While no anatomic change is definitively predictive for all eyes, some features on OCT/OCTA have been identified as potential biomarkers for an increased risk of near-term exudation and may warrant more frequent follow-up:

Shallow Irregular Retinal Pigment Epithelial Elevation (SIRE) or Double-Layer Sign (DLS): These are structural OCT findings that correlate with Type 1 MNV and have been associated with a higher risk of conversion.

Growth in MNV size or changes in vascular morphology on OCTA.

In summary, the key is early detection of the nonexudative MNV via OCTA to identify the high-risk eye, followed by vigilant monitoring for the first signs of exudation, at which point anti-VEGF treatment should be promptly initiated.

### **Grok 4**

Nonexudative macular neovascularization (MNV), also known as subclinical or quiescent MNV, in patients with age-related macular degeneration (AMD) is typically managed through observation rather than active intervention, as it is asymptomatic and does not cause immediate visual decline. This condition, often detected via optical coherence tomography angiography (OCTA), carries a significantly elevated risk of progression to exudative (wet) AMD, with conversion rates reported between 20% and 80% over periods ranging from 6 months to 2 years, and a relative risk up to 18 times higher compared to eyes without MNV. Predictors of exudative conversion include lesion enlargement, vascular network growth, and morphological changes like new capillary loops, though lesion size alone may not correlate strongly.

Prophylactic treatment with anti-vascular endothelial growth factor (anti-VEGF) agents is not recommended, as clinical trials like the PRO-CON study have shown no significant benefit in preventing exudation (e.g., conversion rates of 6-31% in treated vs. sham groups over 24 months), and such therapy could potentially accelerate geographic atrophy (GA) by disrupting vessels that may provide nutritional support to the retinal pigment epithelium (RPE) and photoreceptors. In fact, nonexudative MNV may have a protective role against GA progression in some cases, with slower atrophy growth rates observed in affected eyes.

Instead, management emphasizes close surveillance to detect early signs of exudation, allowing for prompt initiation of standard anti-VEGF therapy if fluid develops on structural OCT. Monitoring strategies include:

Frequent imaging: Serial OCTA (preferably swept-source for better sensitivity and visualization) combined with structural OCT to track MNV changes and identify precursors like the double-layer sign or shallow irregular RPE elevation.

Follow-up intervals: More intensive clinic visits (e.g., every 3-6 months) for high-risk patients, such as those with MNV in the fellow eye of unilateral wet AMD or concurrent GA.

Home monitoring and emerging tools: Patient self-monitoring for symptoms, supplemented by home OCT devices or AI-based screening for structural signs, though these require further validation.

General AMD care, such as antioxidant supplements (e.g., AREDS2 formula) for intermediate dry AMD, lifestyle modifications (smoking cessation, UV protection), and regular visual acuity screening, should continue as part of holistic management. There are no formal society guidelines specifically for nonexudative MNV, but decisions should be individualized based on patient age, comorbidities, and access to care, balancing the burden of frequent monitoring against the risk of vision loss from delayed intervention.

### **Claude-Sonnet 4.5**

The management of nonexudative (or "quiescent") macular neovascularization (MNV) detected by OCTA in AMD patients is an evolving area. Here's the current approach:

Observation vs Treatment

Current Standard: Close Monitoring

Most nonexudative MNV is managed with observation rather than immediate anti-VEGF treatment

These lesions represent type 1 (sub-RPE) neovascularization without fluid on OCT

Studies show conversion rates to exudative AMD of approximately 30-50% over 2-3 years

Monitoring Protocol:

OCT examination every 3-4 months initially

Patient education about home Amsler grid monitoring

Watch for development of:

Intraretinal fluid (IRF)

Subretinal fluid (SRF)

Pigment epithelial detachment (PED) enlargement

Visual symptoms

Treatment Considerations

Treat when exudation develops:

Initiate anti-VEGF therapy when fluid appears on OCT

Standard loading dose protocol (typically 3 monthly injections)

Prophylactic treatment (controversial):

Some evidence suggests early treatment may preserve vision better

PREVENTION study and ongoing trials are evaluating this approach

Not yet standard of care

Risk Stratification

Higher-risk lesions that warrant closer monitoring:

Larger lesion area

Presence of subretinal hyperreflective material

Progressive growth on serial imaging

Fellow eye with exudative AMD

The key is balancing the risk of conversion against the burden and risks of unnecessary anti-VEGF injections. Would you like more details about any specific aspect of this management approach?

### **Qwen3-Max**

The management of nonexudative macular neovascularization (MNV)—also referred to as quiescent or subclinical MNV—detected by optical coherence tomography angiography (OCTA) in patients with age-related macular degeneration (AMD) remains an evolving area. Current evidence supports a monitoring-based approach rather than immediate treatment, as these lesions are asymptomatic and lack signs of exudation (e.g., fluid, hemorrhage, or vision loss).

Key Management Principles:

1. Close Monitoring:

Nonexudative MNV is typically managed with regular follow-up using multimodal imaging, especially OCTA and structural OCT, to detect conversion to exudative (wet) AMD. Studies show that OCTA enables non-invasive, contrast-free, and repeatable monitoring of MNV progression .

2. Risk of Conversion:

Research indicates that growing MNV on OCTA is associated with a higher probability and faster onset of exudative conversion, suggesting that quantitative OCTA metrics may help stratify risk .

3. No Routine Anti-VEGF Treatment:

There is no current consensus supporting prophylactic anti-VEGF therapy for nonexudative MNV. Treatments like ranibizumab or aflibercept are reserved for exudative complications .

4. Imaging Advances Aid Detection and Follow-up:

Swept-source OCTA (SS-OCTA) enhances detection and allows both qualitative and quantitative evaluation of nonexudative MNV, improving early identification and management decisions .

5. Emerging Tools:

Deep learning models using OCT B-scans are being developed to assist in detecting nonexudative MNV, potentially streamlining screening in clinical practice .

Summary:

Nonexudative MNV in AMD should be monitored closely with OCTA and OCT, with treatment initiated only upon signs of exudation or visual decline. Proactive imaging helps identify high-risk features (e.g., lesion growth) that may herald conversion to exudative disease .

# **S4. Notes on Full Raw Outputs**

The representative outputs above are provided to improve reproducibility while keeping the supplementary appendix manageable. The original model-specific raw-output files contain the full responses for the 30 prompts across the six evaluated models. Additional raw outputs are available from the corresponding author on reasonable request or may be uploaded separately according to journal requirements.
